# Supplementary material for: Clinical practice guidelines for cardiovascular disease: how is depression addressed? Protocol for a systematic review
Source: BMJ Open. 2023 May 2;13(5):e071940. doi: 10.1136/bmjopen-2023-071940 (PMC10163515; doi:10.1136/bmjopen-2023-071940)
Supplement: Supplementary data [file bmjopen-2023-071940supp002.pdf]

Supplementary File 2: Search strategy for Pubmed using the E-utilities

The search on Pubmed was done by running the E-utilities software (1) from NCBI in a shell script (see below “retrieve_guidelines_pubmed.sh”) developed specifically for this project. The combination of the search results into excel tables was done with the R software and two excel related R-packages (see below “CVD_guidelines_search.R”) (2,3,4)

The keyword strings used in this search were:

“CVD cardiovascular diseases”
“HF heart failure”
“CAD coronary artery disease”
“CVA cerebrovascular accident stroke”
“PAD peripheral artery disease”
“Aortic disease”
“diabetes”
“dyslipidemia”
“HT hypertension”

Pubmed search term per keyword string:

(((guideline[Title]) OR guideline[Publication Type]) AND (keywordString)) AND (("2012"[Date - Publication] : "2023/01/05"Date - Publication]))

Example: For the keyword string “CAD coronary artery disease “, The search query would be:

(((guideline[Title]) OR (guideline[Publication Type])) AND (CAD coronary artery disease)) AND (("2012"[Date - Publication] : "2023/01/05"[Date - Publication]))

Esearch command for each keyword string:

esearch -db pubmed -query "((guideline[Title]) OR guideline[Publication Type]) AND ($KeywordString)" -datetype PDAT -mindate 2012 -maxdate 2023/01/05

This is the equivalent of the manual pubmed search automatized in a bash code (mac-zsh). Only pmids are retrieved using efetch and the parameter: “-format uid”.
Pmids are then sorted for uniqueness using bash. The code then loops over all unique pmids to store publication details into tab-delimited text files using efetch and xtract commands.

Find the original codes attached to this document:

1. *“retrieve_guidelines_pubmed.sh”*
2. *“CVD_guidelines_search.R”*

Code 1: retrieve_guidelines_pubmed.sh

```
#!/bin/zsh

#this code works best on macintosh zsh terminal, to try it on another
shell, replace #!/bin/zsh by #!/bin/sh in the shebang line 1.

#$1 is a text file with keywords strings like "HF heart failure"
#$2 is the start year and $3 is the end year for the search formats:
YYYY or YYYY/MM or YYYY/MM/DD

#lauch code command for 2012-2023/01/05 full search:
#./retrieve_on_keyword_opti.sh keywords.txt 2012 2023/01/05

#the code will create and fill up a set of text and csv files for the
search results.

Lines=$(cat $1|wc -l|sed 's/ //g' |cut -f1) #number of keywords
Date1=$(echo $2 | sed 's/\//_/g'|cut -f1) #reformat dates with
underscores
Date2=$(echo $3 | sed 's/\//_/g'|cut -f1)

PMIDsfile=PMIDs_allkeywords_"$Date1"_to_"$Date2".txt
PMIDKEYSfile=pmid_keys_"$Date1"_to_"$Date2".csv
cat pmid_keys_header.csv > $PMIDKEYSfile

for l in {1..$Lines} #for loop over all keyword strings
do
    Keyword=$(cat $1| head -$l | tail -1)

    echo $l: $Keyword
    if [ -f "$Keyword"_"$Date1"_to_"$Date2"_pmids.txt ];
    then
        rm "$Keyword"_"$Date1"_to_"$Date2"_pmids.txt
    fi
    #from the initial esearch, only pmids are stored into a text file
    esearch -db pubmed -query "((guideline[Title]) OR
guideline[Publication Type]) AND ($Keyword)" -datetype PDAT -mindate $2
-maxdate $3| efetch -format uid
>>"$Keyword"_"$Date1"_to_"$Date2"_pmids.txt

    cat "$Keyword"_"$Date1"_to_"$Date2"_pmids.txt >>tmp1.txt

    for k in $(cat "$Keyword"_"$Date1"_to_"$Date2"_pmids.txt)
    do
        echo "$k,$Keyword" >> tmp_pmid_keys.csv
    done
done

#pmids are now sorted for uniqueness
cat tmp1.txt |cut -f1 | sort -dhu | awk 'NR>1'>tmp2.txt
cat tmp_pmid_keys.csv| sort -k1,1 -u | awk 'NR>1'>>$PMIDKEYSfile
cat tmp2.txt | sort -dhu | awk 'NR>1'>$PMIDsfile
rm tmp1.txt
rm tmp2.txt
```

```
rm tmp_pmid_keys.csv

#step2

Detailsfile=Details_Publications_"$Date1"_to_"$Date2".txt
DOIsfile=DOIs_Publications_"$Date1"_to_"$Date2".txt
Abstractsfile=Abstracts_Publications_"$Date1"_to_"$Date2".txt
Typefile=Pub_types_"$Date1"_to_"$Date2".txt

cat header_opti.txt >$Detailsfile
cat DOIheader.txt >$DOIsfile
cat Typeheader.txt >$Typefile
cat abstractsheader.txt >$Abstractsfile
pLines=$(cat $PMIDsfile|wc -l|sed 's/ //g' |cut -f1)

echo "running for $pLines entries"

for p in {1..$pLines} #for loop over all unique pmid to store
publication details into tab delimited text files
do
    PMID=$(cat $PMIDsfile| head -$p | tail -1)

    if [ $(expr $p % 50) -eq 0 ]
    then
        echo "$p over $pLines"
    fi
    #efetch efficiency depends a lot on internet connexion speed and
stability
     efetch -db pubmed -id $PMID -format xml > temp.xml
    cat temp.xml| xtract -pattern PubmedArticle -def "-" -element
MedlineCitation/PMID PubDate/Year PubDate/Month PubDate/Day
PubDate/MedlineDate DateRevised/Month Article/ArticleTitle
Article/VernacularTitle Journal/Title JournalIssue/Volume
JournalIssue/Issue Pagination/MedlinePgn Abstract
MedlineJournalInfo/Country -block Author -tab ", "  -sep " " -element
LastName,Initials >> $Detailsfile

    cat temp.xml | xtract -pattern PubmedArticle -def "-" -element
MedlineCitation/PMID -block Article -tab "; "  -sep "; " -element
ELocationID@EIdType ELocationID >>$DOIsfile

    cat temp.xml | xtract -pattern PubmedArticle -def "-" -element
MedlineCitation/PMID -block Article -tab "; "  -sep "; " -element
PublicationTypeList/PublicationType >>$Typefile

     echo "$(cat temp.xml | xtract -pattern PubmedArticle -def "-" -
element MedlineCitation/PMID -block Article -tab "; "  -sep "; " -def
"-"  -element Abstract/AbstractText | tr -d '\n')\n" >>$Abstractsfile
done

echo "its over";
rm temp.xml
```

Code 2: CVD_guidelines_search.R

```
#You must first run the shell script "retrieve_guidelines_pubmed.sh"

library(xlsx)
library(readxl)

fileversion<-"_v06"
period<-"2012_to_2023/05/01"
filesLocation<-"~/Documents/oneshots/Litterature_search/"

pubmed<-
read.delim(paste0(filesLocation,"Details_Publications_",period,".txt"))
dois<-
read.delim(paste0(filesLocation,"DOIs_Publications_",period,".txt"))
abstracts<-
read.delim(paste0(filesLocation,"Abstracts_Publications_",period,".txt"
))
keys<-read.csv(paste0(filesLocation,"pmid_keys_",period,".csv"))
types<-read.delim(paste0(filesLocation,"Pub_types_",period,".txt"))

getDOI<-function(Elo){
  a<-paste0(strsplit(Elo,split="; ")[[1]],"")

  if (length(a)==2){
    return(paste0(a[2][a[1]=="doi"][1],""))
  }
  if (length(a)>2){
    b<-matrix(a,ncol=2)
    return(paste0(b[,2][b[,1]=="doi"][1],""))
  }
  else{
    return ("")}
}

dois$DOI<-sapply(dois$ELocationIDs, function(x) getDOI(x))
dois$DOI[dois$DOI!=""]<-paste0("http://doi.org/",dois$DOI[dois$DOI!=""
&!is.na(dois$DOI)])

abstracts$depression<-sapply(abstracts$Abstract,function(x)
grepl("depress",x,ignore.case = T))
abstracts$psych<-sapply(abstracts$Abstract,function(x)
grepl("psych",x,ignore.case = T))
abstracts$mental<-sapply(abstracts$Abstract,function(x) grepl(" mental
",x,ignore.case = T)|grepl(" mentally ",x,ignore.case = T))

pubmed$keywords<-
sapply(pubmed$PMID,function(x)paste(keys$Keyword[keys$PMID==x],collapse
=", "))

if(identical(pubmed$PMID,dois$PMID)){
  pubmed$DOI<-dois$DOI
  pubmed$DOI[pubmed$DOI=="http://doi.org/NA"]<-""
}
```

```
if(identical(pubmed$PMID,abstracts$PMID)){
  pubmed$depression<-abstracts$depression
  pubmed$psych<-abstracts$psych
  pubmed$mental<-abstracts$mental
  pubmed$Abstract[abstracts$Abstract!=""]<-"accessible"
}
if(identical(pubmed$PMID,types$PMID)){
  pubmed$Publication_types<-types$Publication_types
}

pubmed[,"Relev/NR = 1/0 ?"]<-character(nrow(pubmed))
pubmed<-pubmed[,c(1:6,ncol(pubmed),7:(ncol(pubmed)-1))]

write.xlsx(pubmed,paste0(filesLocation,"litterature_on_CVD_and_MH_Every
thing_",period,fileversion,".xlsx"),sheetName =
paste0("Pubmed_",period),showNA = F,row.names = F)
write.xlsx(pubmed[pubmed$depression|pubmed$psych|pubmed$mental,],paste0
(filesLocation,"litterature_on_CVD_and_MH_Everything_",period,fileversi
on,".xlsx"),sheetName = paste0("Pubmed_PSY",period),showNA =
F,row.names = F,append = T)

selection<-c("PMID",   "Year",     "Relev/NR = 1/0 ?",    "Title",
      "Journal",  "DOI",      "Publication_types")

write.xlsx(pubmed[,selection][grepl("guideline",pubmed$Publication_type
s, ignore.case = T) & pubmed$VernacularTitle=="-
",],paste0(filesLocation,"litterature_on_CVD_trimed_",period,fileversio
n,".xlsx"),sheetName = paste0("Guidelines_PubType_ENG"),showNA =
F,row.names = F)
write.xlsx(pubmed[,selection][!grepl("guideline",pubmed$Publication_typ
es, ignore.case = T) & pubmed$VernacularTitle=="-
",],paste0(filesLocation,"litterature_on_CVD_trimed_",period,fileversio
n,".xlsx"),sheetName = paste0("Guidelines_Title_ENG"),showNA =
F,row.names = F,append=T)
write.xlsx(pubmed[,selection][pubmed$VernacularTitle!="-
",],paste0(filesLocation,"litterature_on_CVD_trimed_",period,fileversio
n,".xlsx"),sheetName = paste0("non_English_pubs",showNA = F,row.names =
F,append=T)
```

References

1. Sayers E. The E-utilities In-Depth: Parameters, Syntax and More. 2009 May 29 [Updated 2022 Nov 30]. In: Entrez Programming Utilities Help [Internet]. Bethesda (MD): National Center for Biotechnology Information (US); 2010-. Available from: https://www.ncbi.nlm.nih.gov/books/NBK25499/

2. R Core Team (2022). R: A language and environment for statistical computing. R Foundation for Statistical Computing, Vienna, Austria. URL https://www.R-project.org/

3. Dragulescu A, Arendt C (2020). _xlsx: Read, Write, Format Excel 2007 and Excel 97/2000/XP/2003 Files_. R package version 0.6.5, https://CRAN.R-project.org/package=xlsx

4. Wickham H, Bryan J (2022). _readxl: Read Excel Files_. R package version 1.4.1, https://CRAN.R-project.org/package=readxl
