# Supplementary material for: Clinical practice guidelines for cardiovascular disease: how is depression addressed? Protocol for a systematic review
Source: BMJ Open. 2023 May 2;13(5):e071940. doi: 10.1136/bmjopen-2023-071940 (PMC10163515; doi:10.1136/bmjopen-2023-071940)
Supplement: Author's manuscript [file bmjopen-2023-071940.draft_revisions.pdf]

BMJ Open is committed to open peer review. As part of this commitment we make the peer review history of every article we publish publicly available.

When an article is published we post the peer reviewers' comments and the authors' responses online. We also post the versions of the paper that were used during peer review. These are the versions that the peer review comments apply to.

The versions of the paper that follow are the versions that were submitted during the peer review process. They are not the versions of record or the final published versions. They should not be cited or distributed as the published version of this manuscript.

BMJ Open is an open access journal and the full, final, typeset and author-corrected version of record of the manuscript is available on our site with no access controls, subscription charges or pay-per-view fees (http://bmjopen.bmj.com).

If you have any questions on BMJ Open's open peer review process please email info.bmjopen@bmj.com

BMJ Open

Clinical practice guidelines for cardiovascular disease: how is depression addressed? Protocol for a systematic review

Journal:	*BMJ Open*
Manuscript ID	bmjopen-2023-071940
Article Type:	Protocol
Date Submitted by the Author:	25-Jan-2023

Complete List of Authors:	Blatch Armon, Dana; International Center for Multimorbidity and Complexity in Medicine, University of Zurich, University Hospital Basel (Department of Psychosomatic Medicine), Merian Iselin Klinik, Basel, Switzerland<br>Buhayer, Aliki; International Center for Multimorbidity and Complexity in Medicine, University of Zurich, University Hospital Basel (Department of Psychosomatic Medicine), Merian Iselin Klinik, Basel, Switzerland<br>Dobretz, Kevin; Geneva University Hospitals, Cardiology<br>Meinlschmidt, Gunther; University Hospital Basel and University of Basel, Department of Psychosomatic Medicine; Cognitive Behavioural Therapy International Psychoanalytic University<br>Battegay, Edouard; International Center for Multimorbidity and Complexity in Medicine, University of Zurich, University Hospital Basel (Department of Psychosomatic Medicine), Merian Iselin Klinik, Basel, Switzerland; University Hospital Basel, Psychosomatic Medicine

Keywords:	CARDIOLOGY, Depression & mood disorders < PSYCHIATRY, MENTAL HEALTH, PRIMARY CARE

SCHOLARONE™
Manuscripts

Clinical practice guidelines for cardiovascular disease: how is depression addressed? Protocol for a systematic review

Blatch Armon Dana*[1], Buhayer Aliki*[1], Dobretz Kevin[2], Meinlschmidt Gunther[3,4], Battegay Edouard[1,5]

Correspondence to: Dr Dana Blatch Armon; dana.blatch@mail.huji.ac.il
Merian Iselin Klinik, Föhrenstrasse 2, 4009 Basel, Switzerland

Author affiliations

* Contributed equally to this paper
1 International Center for Multimorbidity and Complexity in Medicine, University of Zurich, University Hospital Basel (Department of Psychosomatic Medicine), Merian Iselin Klinik, Basel, Switzerland
2 Geneva University Hospitals, Department of Cardiology, Geneve, Switzerland
3 Department of Psychosomatic Medicine, University Hospital Basel and University of Basel, Basel, Switzerland
4 Division of Clinical Psychology and Cognitive Behavioural Therapy, International Psychoanalytic University (IPU) Berlin, Berlin, Germany
5 University Hospital Basel, Department of Psychosomatic Medicine, Basel, Switzerland

Emails of all authors
dana.blatch@mail.huji.ac.il
ab@prismscientific.ch
Kevin.Dobretz@hcuge.ch
gunther.meinlschmidt@unibas.ch
edouard.battegay@uzh.ch

Orcid authors:
Dana https://orcid.org/0000-0002-2160-9144
Aliki https://orcid.org/0000-0002-6538-0273
Edouard https://orcid.org/0000-0001-9202-5034
Gunther https://orcid.org/0000-0002-3488-193X

Word count: 1757

For peer review only

Abstract

Introduction

Depression frequently affects patients with cardiovascular disease (CVD). When these conditions co-occur, outcomes such as quality of life and life expectancy worsen. In everyday practice, this specific and prevalent disease-disease interaction complicates patient management. Clinical practice guidelines (CPG) aim to provide the best available advice for clinical decision-making to improve patient care. This study will aim to evaluate how CPGs specifically address depression in patients with CVD, and whether they provide any operational guidance for screening for and management of depression in the primary care setting.

Methods and analysis

We will conduct a systematic review of clinical practice guidelines on CVD management, published from 2012 to 2023. A broad literature search for guidelines will be performed through electronic medical databases, grey literature search tools, and websites of national and professional medical organizations.
Based on the inclusion criteria, eligible guidelines will be assessed for recommendations on depression management in patients with CVD. Additional points to be evaluated will be any mention of drug-drug or drug-disease interactions, other aspects specific to treating physicians, as well as general information on mental health. We will assess the quality of CPGs with a recommendation regarding depression in CVD patients using the Appraisal of Guidelines for Research and Evaluation II (AGREE-II).

Ethics and dissemination

As this systematic review is based on available published data, ethics approval and consent are not applicable. We intend that our results will be published in a peer-reviewed journal, presented at international scientific meetings, and distributed to healthcare providers.

PROSPERO registration number CRD42022384152

Article summary

Strengths and limitations of this study

- The rigorous literature search method we developed aims to produce an extensive record of CVD CPGs, to accurately describe how depression screening and management is addressed in these patients.
- Our literature search will cover the past 10 years to provide insights into current and clinically relevant guidelines, while also facilitating the identification of possible trends in recommendations.

For peer review only - http://bmjopen.bmj.com/site/about/guidelines.xhtml

- This systematic review will use the Appraisal of Guidelines for Research & Evaluation (AGREE) II instrument to evaluate the quality of the selected guidelines, including all relevant recommendations.
- This study will include only CPGs published in English, excluding any guidelines produced in other languages.
- Focusing on primary care might limit the applicability of findings to other settings. However, the majority of the management of patients with CVD and depression takes place in primary care.

Introduction

Despite substantial advances in the field, cardiovascular disease (CVD) is still the leading cause of morbidity and mortality in the world (1). Improvements in primary prevention and management of risk factors have lowered the risk of cardiovascular events and reduced age-specific mortality over the past decades (1,2). However, higher life expectancy has led to a rise in comorbid conditions in CVD patients (3).

Multimorbidity, commonly described as the occurrence of two or more chronic conditions, in one individual (4), is associated with increased mortality, decreased quality of life, and increased use of healthcare services (3,5,6), including visits to general practitioners and other medical specialists (7). In addition, multimorbidity leads to challenging situations in the treatment and management of these patients, further complicating medical decision-making (7,8). The relationship between CVD and multimorbidity is well established (9–11), and among multimorbidities, depression is one of the most common co-occurring conditions (12).

Depression is a common mental disorder that interferes with a person's mood and behavior.(13). It affects up to 10% of adults throughout their lifetime (14) and is considered to be the leading cause of disability worldwide (15). In patients with CVD, the occurrence of depression is estimated to be 1 in 5 (12). Depression has also been associated with increased incidence and worse outcomes of coronary artery disease (CAD). People with depressive disorders (DD) are also more prone to develop acute myocardial infarction (AMI), heart failure (HF), and stroke (16,17). When controlling for traditional CVD risk factors, the presence of depressive symptoms even without a clinical diagnosis of DD can predict the incidence of CAD in healthy individuals (18), as well as secondary events in patients with CAD (19–22), and adverse outcomes following coronary artery bypass grafting (23).

In patients with CVD, depression was also associated with an elevated risk of failure to adhere to medications and a healthy lifestyle (24,25). patients with depression may be less likely to engage in behaviors that lower the risk of future cardiovascular events, such as physical activity, dietary modifications, smoking cessation, stress management, and treatments against substance abuse (26,27).

Inadequate depression treatment is associated with a higher risk of adverse cardiovascular events (28). Primary care physicians play an important role in recognizing and managing depression, in particular since an estimated 60% of mental health care delivery occurs in the primary care setting (29). Despite high evidence on the importance of diagnosis and treatment of depression (30), implementation in practice remains limited (31), and over 50% of patients with depression in primary care remain unrecognized and inadequately treated (32–35).

For peer review only - http://bmjopen.bmj.com/site/about/guidelines.xhtml

Clinical practice guidelines (CPG) provide the best available evidence to support clinical decision-making and aim to improve care and outcomes (36), however, they vary in their recommendations regarding screening for depression. For example, the US Preventive Services Task Force (USPSTF) recommends screening for depression in the general adult population (37), whereas official guidelines by the National Institute for Health and Clinical Excellence (NICE), as well as The Canadian Task Force on Preventive Health Care (CTFPHC) recommend not to screen routinely for depression in the primary care setting (38,39).
A recent state-of-the-art review on depression in patients with CVD summarized selected guidelines on screening for depression and showed that recommendations are only partially available and inconsistent (40). The recommendations tended to focus on specific sub-patient groups and lacked a systematic approach, also regarding which screening tools to use, leaving an incomplete picture of practical recommendations for practitioners to follow in their daily practice (41).

Evidence gap

The systematic review of existing CPGs is an approach that is increasingly being used in an attempt to characterize the nature of clinical guidance on a topic of interest (36). To our knowledge, no such review has yet been performed regarding how depression is addressed in guidelines on CVD. As standardized screening and treatment pathways offer the potential for early identification and improved management of both CVD and depression (42–44), we aim to conduct such a review in order to provide a clear picture of the recommendations currently available and identify gaps in knowledge. This step is crucial to provide guidance for future research and, ultimately, for improving the quality of evidence-based clinical recommendations.

Aim

The aim of this systematic review will be to identify, appraise and describe how CPGs for CVD management specifically address depression, and whether they provide operational guidance for screening and management to practitioners.
This systematic review is part of a larger review currently in process that is meant to provide a comprehensive picture of knowledge and evidence gathered so far on patients with concurrent CVD and depression. It is also intended to identify knowledge gaps in the current state of clinical guidance, thereby promoting further research to produce evidence-based guidance for future guidelines to further improve treatment for patients.

Objectives

1. Identify published CPGs on the management of CVD in the primary care setting.
2. Among all qualifying CPGs on CVD, identify those that mention and provide recommendations for depression as comorbidity or multimorbidity.
3. Assess the quality of recommendations using the Appraisal of Guidelines for Research and Evaluation version 2 (AGREE II) instrument.

For peer review only - http://bmjopen.bmj.com/site/about/guidelines.xhtml

4. Summarize the current situation with regard to the consideration of depression in CPG on CVD.

Methods and analysis

For the preparation of this protocol we followed specific methodological guidance for conducting systematic reviews of CPGs, as such reviews require a tailored approach to design and execution compared with other systematic reviews (36). This protocol follows the PRISMA-P guidelines for the reporting of systematic review protocols (45). The completed checklist for this protocol is available in the Supplementary File 1.

Patient and public involvement

Patients and/or the public were not involved in the design, or conduct, or reporting, or dissemination plans of this research protocol.

Eligibility criteria

The PICAR (46) framework was used to guide inclusion and exclusion criteria and is described in Table 1. These selected guidelines will be assessed for recommendations regarding depression in patients with CVD. We will use the Preferred Reporting Items for Systematic Reviews and Meta-Analyses guidelines (PRISMA) to report the results of this review (47).

Table 1 PICAR statement

P: Population, clinical indication(s) and condition(s)	Study population: adults 18 and above with CVD in the primary care setting<br>Clinical indication: Management of patients with CVD<br>Clinical condition: CVD
I: Intervention(s)	Any intervention focusing on diagnosis and/or treatment of depression
C: Comparator(s)	No comparator. All aspects of depression diagnosis and management will be taken into consideration.
A: Attributes of eligible CPGs	• Language: English<br>• Time range: 1st Jan 2012 until 5th January 2023. CPGs that were published before 2012 and have not been renewed or updated since were considered out of date on the basis of the rapid advances in the field of CVD over the past decade.<br>• Publishing region: no restriction<br>• Versions: latest versions only<br>• Developing publishing organization: endorsed by national or international scientific societies, professional colleges,

For peer review only

	charitable organizations, and government organizations will be included<br>● Publication status: only published CPGs.<br>● Development process: explicitly evidence- and/or consensus-based<br>● System of rating evidence: Use of a system to rate the level of evidence within CPGs is not an eligibility criterion; but will be taken into consideration when available and stated clearly<br>● Scope: CPGs for the general management of adult patients with CVD (including heart failure, coronary artery disease, stroke, and hypertension management) in the community setting.
R: Recommendations characteristics	Recommendations covering screening, diagnosis, and pharmacological and non-pharmacological management of depression are of interest.

CVD cardiovascular disease, CPG clinical practice guidelines

Search strategy and information sources

To identify all relevant CPGs, the search strategy was developed on the basis of the Johnston paper recommendations for a systematic review of CPGs and under the guidance of an experienced bioinformatician (KD). We will conduct a systematic search in the following databases: PUBMED, TRIP, EMBASE, Guidelines international network (GIN), and Guideline central, using search terms and methods adjusted to the specificities of each database (example in Supplementary file 2). In order to locate additional relevant CPGs not available in academic journals, we will extend our search range to include additional grey literature that will target guidelines by specifically searching on the Society guideline link on Uptodate, World Health Organization guidelines section, and the websites of national and professional medical organizations (e.g., National Institute for Health and Care Excellence (NICE), Scottish Intercollegiate Guidelines Network (SIGN)).

Guideline selection

All results collected in the literature search will be summarized in a table and submitted to two independent reviewers (DB and AB) who will conduct a two-step selection procedure for eligibility, based on the pre-defined inclusion and exclusion criteria:

1. First step - Titles and abstracts will be screened for relevance. Duplicates will be removed in this step. Potentially relevant CPGs where an abstract is not provided will be passed to the second screening stage.
2. Second step - full-text screening to assess CPGs for final inclusion in the review.

Records will be excluded if both reviewers agreed they were not eligible. Any disagreements will be resolved by consensus. If consensus cannot be met between the two reviewers, a third reviewer (EB) will also assess the particular record to determine its eligibility.

For peer review only - http://bmjopen.bmj.com/site/about/guidelines.xhtml

Inclusion criteria

1. CPGs for the general management of adult patients with CVD (including specific guidelines for heart failure, coronary artery disease, stroke, and hypertension management) in the community setting.
2. The CPG is published in English.
3. The CPG was published between 2012 and 5th January 2023.
4. The CPG is the most recent version.
5. The full CPG is available online.
6. The CPG was written by national and international academic organizations.

Exclusion criteria

1. Guidelines limited to a specific aspect of CVD management, such as screening, pharmacological management, specific interventions, acute setting, or hospital setting, not relevant to primary care practice.
2. Target population under 18 years of age.

Data extraction and analysis

Data management and extraction

Once the final set of included CPGs has been obtained, DB and AB will conduct a search in the full text for any mention of depression, using the search terms “depress” “mental” “psych” and “mood”.

First, all results collected will be summarized in a table containing the CPG title, year of publication, organization and country, and the exact context in the document (page number, chapter, line, table, or figure number).

Second, DB and AB will assess and categorize each result for any operational guidance on screening and/or management of depression in patients with CVD. Any mention of interactions and points of consideration for treating physicians, as well as general information on mental health issues will also be included.

Specifically, the following aspects will be examined and classified:

- Screening recommendation: population, how to screen (who, what screening tool), when (and how often), and what to do with a positive screening result (treatment, referral).
- Management - pharmacological and non-pharmacological treatment modalities, drug-drug or drug-disease interactions to consider.

Special consideration will be given to the level of evidence on which the recommendation is based, and will be reported when available. In addition, the quality of the selected CPGs will be assessed using the updated AGREE-II instrument (48). As the study progresses, the datasheet may evolve to fit the study’s objectives better. The full list of variables of interest is shown in Table 2.

Table 2 List of variables

For peer review only - http://bmjopen.bmj.com/site/about/guidelines.xhtml

Name of variable
CPG topic (CVD or specific condition i.e., HTN, HF, CAD, stroke)
CPG title
Organization/society responsible for the guideline
Year of publication
Source of funding
Country
Grading system for recommendations
Depression, any mention
Depression screening and management, any recommendation
CVD and depression interactions (drug-drug, disease-drug)
Level of evidence of depression recommendation
Overall AGREE-II score of CPG

HTN, hypertension; HF, heart failure; CAD, coronary artery disease
AGREE-II, Appraisal of Guidelines for Research and Evaluation II.

Results

The systematic review will aim at providing results for the following planned steps:

1. A flowchart of the search strategy.
2. A detailed summary of available recommendations (any type) on depression listed by identified CPG on CVD.
3. Quality assessment sheets of CPGs using the AGREE-II methodology.

Significance of the study

Currently, depression in patients with CVD is poorly addressed at the primary care level. This study aims at improving the management of these patients. The first step towards this goal is to identify gaps in knowledge. By evaluating the current state of recommendations available and identifying the gaps in evidence, this study may serve as a guide for tailoring future research in this field. This step is crucial for the development of evidence-based recommendations for future CPGs aiming at providing better guidance on the management of depression in patients with CVD.

For peer review only - http://bmjopen.bmj.com/site/about/guidelines.xhtml

Footnotes

Ethics and dissemination

As this study is a systematic review, a secondary analysis of published data will be performed, and no human participants involved in this research. Therefore, in this situation ethics approval and consent are not applicable.

This study is registered on the International Prospective Register of Systematic Reviews (PROSPERO) as of 26 December 2022 (registration number: CRD42022384152) and will be updated as needed and as main results will be published.

The results of this study will be reported in accordance with the PRISMA statement (47) and disseminated through publication in a peer-reviewed journal, presentation at international scientific conferences, and contact with medical associations responsible for writing guidelines.

Author contributions

EB, AB and DB were involved in the conceptualization of the study. EB, AB, DB and GM designed the protocol and developed the methodology for the literature search strategy. KD will conduct the literature search. DB and AB will conduct the data analysis, quality assessment, and formal analysis. EB will supervise the study and is in charge of Funding acquisition. All authors have made important intellectual content contributions to the protocol, and reviewed and edited its final version.

Funding

None declared for AB and DB and KD.
EB received a nonrestricted educational and research grant of the Zurich Academy of Internal Medicine, https://www.my-zaim.ch.
GM received funding from the Stanley Thomas Johnson Stiftung & Gottfried und Julia Bangerter-Rhyner-Stiftung under projects no. PC 28/17 and PC 05/18, from Gesundheitsförderung Schweiz under project no. 18.191/K50001, from the Swiss Heart Foundation under project no. FF21101, from the Research Foundation of the International Psychoanalytic University (IPU) Berlin, from the Swiss National Science Foundation (SNSF) under project no. 100014_135328, in the context of a Horizon Europe project from the Swiss State Secretariat for Education, Research and Innovation (SERI) under contract number 22.00094, and from Wings Health Inc. in the context of a proof-of-concept study. GM is co-founder, member of the board, and shareholder of Therayou AG, which is active in the field of digital and blended mental healthcare.

For peer review only - http://bmjopen.bmj.com/site/about/guidelines.xhtml

Competing interests

None declared.

References

1. Capewell S, O'Flaherty M. Rapid mortality falls after risk-factor changes in populations. Lancet Lond Engl. 2011 Aug 27;378(9793):752–3.
2. O'Flaherty M, Buchan I, Capewell S. Contributions of treatment and lifestyle to declining CVD mortality: why have CVD mortality rates declined so much since the 1960s? Heart Br Card Soc. 2013 Feb;99(3):159–62.
3. van den Akker M, Buntinx F, Metsemakers JF, Roos S, Knottnerus JA. Multimorbidity in general practice: prevalence, incidence, and determinants of co-occurring chronic and recurrent diseases. J Clin Epidemiol. 1998 May;51(5):367–75.
4. Harrison C, Fortin M, van den Akker M, Mair F, Calderon-Larranaga A, Boland F, et al. Comorbidity versus multimorbidity: Why it matters. J Multimorb Comorbidity [Internet]. 2021 Jan 1 [cited 2022 Oct 14];11:263355652199399. Available from: http://journals.sagepub.com/doi/10.1177/2633556521993993
5. Pati S, Swain S, Hussain MA, van den Akker M, Metsemakers J, Knottnerus JA, et al. Prevalence and outcomes of multimorbidity in South Asia: a systematic review. BMJ Open. 2015 Oct 7;5(10):e007235.
6. Salisbury C, Johnson L, Purdy S, Valderas JM, Montgomery AA. Epidemiology and impact of multimorbidity in primary care: a retrospective cohort study. Br J Gen Pract J R Coll Gen Pract. 2011 Jan;61(582):e12-21.
7. Starfield B, Lemke KW, Bernhardt T, Foldes SS, Forrest CB, Weiner JP. Comorbidity: implications for the importance of primary care in 'case' management. Ann Fam Med. 2003 Jun;1(1):8–14.
8. Farmer C, Fenu E, O'Flynn N, Guthrie B. Clinical assessment and management of multimorbidity: summary of NICE guidance. BMJ. 2016 Sep 21;354:i4843.
9. Aarts S, van den Akker M, Tan FES, Verhey FRJ, Metsemakers JFM, van Boxtel MPJ. Influence of multimorbidity on cognition in a normal aging population: a 12-year follow-up in the Maastricht Aging Study. Int J Geriatr Psychiatry. 2011 Oct;26(10):1046–53.
10. Bang D, Xu J, Keenan R, Pike V, Lehmann R, Tenner C, et al. Cardiovascular Disease Prevalence in Patients with Osteoarthritis, Gout, or Both. Bull Hosp Jt Dis 2013. 2016 Jun;74(2):113–8.
11. Zander N, Schäfer I, Radtke M, Jacobi A, Heigel H, Augustin M. Dermatological comorbidity in psoriasis: results from a large-scale cohort of employees. Arch Dermatol Res. 2017 Jul;309(5):349–56.
12. Thombs BD, Bass EB, Ford DE, Stewart KJ, Tsilidis KK, Patel U, et al. Prevalence of depression in survivors of acute myocardial infarction: Review of the evidence. J Gen Intern Med [Internet]. 2006 Jan [cited 2022 Sep 7];21(1):30–8. Available from: http://link.springer.com/10.1111/j.1525-1497.2005.00269.x
13. American Psychiatric Association. Diagnostic and Statistical Manual of Mental Disorders [Internet]. Fifth Edition. American Psychiatric Association; 2013 [cited 2022 Dec 27]. Available from: https://psychiatryonline.org/doi/book/10.1176/appi.books.9780890425596
14. Lim GY, Tam WW, Lu Y, Ho CS, Zhang MW, Ho RC. Prevalence of Depression in the Community from 30 Countries between 1994 and 2014. Sci Rep. 2018 Feb 12;8(1):2861.
15. World Health Organization. Depression and other common mental disorders: global health

For peer review only - http://bmjopen.bmj.com/site/about/guidelines.xhtml

estimates [Internet]. Geneva: World Health Organization; 2017. Available from: https://apps.who.int/iris/handle/10665/254610
16. Goldstein BI, Carnethon MR, Matthews KA, McIntyre RS, Miller GE, Raghuveer G, et al. Major Depressive Disorder and Bipolar Disorder Predispose Youth to Accelerated Atherosclerosis and Early Cardiovascular Disease: A Scientific Statement From the American Heart Association. Circulation [Internet]. 2015 Sep 8 [cited 2022 Nov 2];132(10):965–86. Available from: https://www.ahajournals.org/doi/10.1161/CIR.0000000000000229
17. Lichtman JH, Froelicher ES, Blumenthal JA, Carney RM, Doering LV, Frasure-Smith N, et al. Depression as a Risk Factor for Poor Prognosis Among Patients With Acute Coronary Syndrome: Systematic Review and Recommendations: A Scientific Statement From the American Heart Association. Circulation [Internet]. 2014 Mar 25 [cited 2022 Sep 30];129(12):1350–69. Available from: https://www.ahajournals.org/doi/10.1161/CIR.0000000000000019
18. Lett HS, Blumenthal JA, Babyak MA, Sherwood A, Strauman T, Robins C, et al. Depression as a risk factor for coronary artery disease: evidence, mechanisms, and treatment. Psychosom Med. 2004 Jun;66(3):305–15.
19. Stewart R. Depression and cardiovascular morbidity and mortality: cause or consequence? Eur Heart J [Internet]. 2003 Nov [cited 2022 Dec 27];24(22):2027–37. Available from: https://academic.oup.com/eurheartj/article-lookup/doi/10.1016/j.ehj.2003.08.017
20. Barth J, Schumacher M, Herrmann-Lingen C. Depression as a risk factor for mortality in patients with coronary heart disease: a meta-analysis. Psychosom Med. 2004 Dec;66(6):802–13.
21. van Melle JP, de Jonge P, Spijkerman TA, Tijssen JGP, Ormel J, van Veldhuisen DJ, et al. Prognostic association of depression following myocardial infarction with mortality and cardiovascular events: a meta-analysis. Psychosom Med. 2004 Dec;66(6):814–22.
22. Evans DL, Charney DS, Lewis L, Golden RN, Gorman JM, Krishnan KRR, et al. Mood disorders in the medically ill: scientific review and recommendations. Biol Psychiatry. 2005 Aug 1;58(3):175–89.
23. Borowicz LJ, Royall R, Grega M, Selnes O, Lyketsos C, McKhann G. Depression and cardiac morbidity 5 years after coronary artery bypass surgery. Psychosomatics. 2002 Dec;43(6):464–71.
24. Gehi A, Haas D, Pipkin S, Whooley MA. Depression and medication adherence in outpatients with coronary heart disease: findings from the Heart and Soul Study. Arch Intern Med. 2005 Nov 28;165(21):2508–13.
25. Sin NL, Kumar AD, Gehi AK, Whooley MA. Direction of Association Between Depressive Symptoms and Lifestyle Behaviors in Patients with Coronary Heart Disease: the Heart and Soul Study. Ann Behav Med Publ Soc Behav Med. 2016 Aug;50(4):523–32.
26. Tang HYJ, Sayers SL, Weissinger G, Riegel B. The role of depression in medication adherence among heart failure patients. Clin Nurs Res. 2014 Jun;23(3):231–44.
27. Stapelberg NJC, Neumann DL, Shum DHK, McConnell H, Hamilton-Craig I. A topographical map of the causal network of mechanisms underlying the relationship between major depressive disorder and coronary heart disease. Aust N Z J Psychiatry. 2011 May;45(5):351–69.
28. Bangalore S, Shah R, Pappadopulos E, Deshpande CG, Shelbaya A, Prieto R, et al. Cardiovascular hazards of insufficient treatment of depression among patients with known cardiovascular disease: a propensity score adjusted analysis. Eur Heart J Qual Care Clin Outcomes. 2018 Oct 1;4(4):258–66.
29. Frank RG, Huskamp HA, Pincus HA. Aligning incentives in the treatment of depression in primary care with evidence-based practice. Psychiatr Serv Wash DC. 2003 May;54(5):682–7.

For peer review only - http://bmjopen.bmj.com/site/about/guidelines.xhtml

30. Bhattacharjee S, Goldstone L, Vadiei N, Lee JK, Burke WJ. Depression Screening Patterns, Predictors, and Trends Among Adults Without a Depression Diagnosis in Ambulatory Settings in the United States. Psychiatr Serv Wash DC. 2018 Oct 1;69(10):1098–100.
31. Fuster V, Kelly BB, Vedanthan R. Global cardiovascular health: urgent need for an intersectoral approach. J Am Coll Cardiol. 2011 Sep 13;58(12):1208–10.
32. Smithson S, Pignone MP. Screening Adults for Depression in Primary Care. Med Clin North Am [Internet]. 2017 Jul [cited 2022 Sep 30];101(4):807–21. Available from: https://linkinghub.elsevier.com/retrieve/pii/S0025712517300330
33. Trivedi MH, Jha MK, Kahalnik F, Pipes R, Levinson S, Lawson T, et al. VitalSign(6): A Primary Care First (PCP-First) Model for Universal Screening and Measurement-Based Care for Depression. Pharm Basel Switz. 2019 May 14;12(2).
34. Olfson M, Blanco C, Marcus SC. Treatment of Adult Depression in the United States. JAMA Intern Med. 2016 Oct 1;176(10):1482–91.
35. Kato E, Borsky AE, Zuvekas SH, Soni A, Ngo-Metzger Q. Missed Opportunities for Depression Screening and Treatment in the United States. J Am Board Fam Med JABFM. 2018 Jun;31(3):389–97.
36. Johnston A, Kelly SE, Hsieh SC, Skidmore B, Wells GA. Systematic reviews of clinical practice guidelines: a methodological guide. J Clin Epidemiol [Internet]. 2019 Apr [cited 2022 Dec 27];108:64–76. Available from: https://linkinghub.elsevier.com/retrieve/pii/S0895435618302877
37. Siu AL, Bibbins-Domingo K, Grossman DC, Baumann LC, Davidson KW, Ebell M, et al. Screening for Depression in Adults: US Preventive Services Task Force Recommendation Statement. JAMA. 2016 Jan 26;315(4):380–7.
38. National Institute for Health and Care Excellence. Depression in adults: treatment and management [NG222] [Internet]. 2022 [cited 2022 Dec 30]. Available from: https://www.nice.org.uk/guidance/ng222
39. Joffres M, Jaramillo A, Dickinson J, Lewin G, Pottie K, Shaw E, et al. Recommendations on screening for depression in adults. CMAJ Can Med Assoc J Assoc Medicale Can. 2013 Jun 11;185(9):775–82.
40. Jha MK, Qamar A, Vaduganathan M, Charney DS, Murrough JW. Screening and Management of Depression in Patients With Cardiovascular Disease. J Am Coll Cardiol [Internet]. 2019 Apr [cited 2022 Sep 27];73(14):1827–45. Available from: https://linkinghub.elsevier.com/retrieve/pii/S0735109719305133
41. Fischer F, Lange K, Klose K, Greiner W, Kraemer A. Barriers and Strategies in Guideline Implementation-A Scoping Review. Healthc Basel Switz. 2016 Jun 29;4(3).
42. Berkman LF, Blumenthal J, Burg M, Carney RM, Catellier D, Cowan MJ, et al. Effects of treating depression and low perceived social support on clinical events after myocardial infarction: the Enhancing Recovery in Coronary Heart Disease Patients (ENRICHD) Randomized Trial. JAMA. 2003 Jun 18;289(23):3106–16.
43. Glassman AH, O'Connor CM, Califf RM, Swedberg K, Schwartz P, Bigger JTJ, et al. Sertraline treatment of major depression in patients with acute MI or unstable angina. JAMA. 2002 Aug 14;288(6):701–9.
44. Kim JM, Stewart R, Lee YS, Lee HJ, Kim MC, Kim JW, et al. Effect of Escitalopram vs Placebo Treatment for Depression on Long-term Cardiac Outcomes in Patients With Acute Coronary Syndrome: A Randomized Clinical Trial. JAMA. 2018 Jul 24;320(4):350–8.
45. PRISMA-P Group, Moher D, Shamseer L, Clarke M, Ghersi D, Liberati A, et al. Preferred reporting items for systematic review and meta-analysis protocols (PRISMA-P) 2015 statement. Syst Rev [Internet]. 2015 Dec [cited 2022 Dec 30];4(1):1. Available from: https://systematicreviewsjournal.biomedcentral.com/articles/10.1186/2046-4053-4-1
46. Methley AM, Campbell S, Chew-Graham C, McNally R, Cheraghi-Sohi S. PICO, PICOS and SPIDER: a comparison study of specificity and sensitivity in three search tools for qualitative

For peer review only

systematic reviews. BMC Health Serv Res [Internet]. 2014 Dec [cited 2022 Dec 30];14(1):579. Available from: https://bmchealthservres.biomedcentral.com/articles/10.1186/s12913-014-0579-0
47. Page MJ, McKenzie JE, Bossuyt PM, Boutron I, Hoffmann TC, Mulrow CD, et al. The PRISMA 2020 statement: an updated guideline for reporting systematic reviews. BMJ [Internet]. 2021 Mar 29 [cited 2022 Dec 30];n71. Available from: https://www.bmj.com/lookup/doi/10.1136/bmj.n71
48. Brouwers MC, Kerkvliet K, Spithoff K, AGREE Next Steps Consortium. The AGREE Reporting Checklist: a tool to improve reporting of clinical practice guidelines. BMJ [Internet]. 2016 Mar 8 [cited 2022 Dec 30];i1152. Available from: https://www.bmj.com/lookup/doi/10.1136/bmj.i1152

For peer review only

For peer review only - http://bmjopen.bmj.com/site/about/guidelines.xhtml

Supplementary File 1: Preferred Reporting Items for Systematic Review and Meta-Analysis for Protocols (PRISMA-P) checklist

Section and topic	Item No	Checklist item	Our response
**ADMINISTRATIVE INFORMATION**			
Title:			
Identification	1a	Identify the report as a protocol of a systematic review	YES
Update	1b	If the protocol is for an update of a previous systematic review, identify as such	N/A
Registration	2	If registered, provide the name of the registry (such as PROSPERO) and registration number	YES
Authors:			
Contact	3a	Provide name, institutional affiliation, e-mail address of all protocol authors; provide physical mailing address of corresponding author	YES
Contributions	3b	Describe contributions of protocol authors and identify the guarantor of the review	YES
Amendments	4	If the protocol represents an amendment of a previously completed or published protocol, identify as such and list changes; otherwise, state plan for documenting important protocol amendments	N/A
Support:			
Sources	5a	Indicate sources of financial or other support for the review	YES
Sponsor	5b	Provide name for the review funder and/or sponsor	N/A
Role of sponsor or funder	5c	Describe roles of funder(s), sponsor(s), and/or institution(s), if any, in developing the protocol	YES
**INTRODUCTION**			
Rationale	6	Describe the rationale for the review in the context of what is already known	YES
Objectives	7	Provide an explicit statement of the question(s) the review will address with reference to participants, interventions, comparators, and outcomes (PICO)	YES
**METHODS**			
Eligibility criteria	8	Specify the study characteristics (such as PICO, study design, setting, time frame) and report characteristics (such as years considered, language, publication status) to be used as criteria for eligibility for the review	YES
Information sources	9	Describe all intended information sources (such as electronic databases, contact with study authors, trial registers or other grey literature sources) with planned dates of coverage	YES
Search strategy	10	Present draft of search strategy to be used for at least one electronic database, including planned limits, such that it could be repeated	YES

For peer review only - http://bmjopen.bmj.com/site/about/guidelines.xhtml

Study records:			
Data management	11a	Describe the mechanism(s) that will be used to manage records and data throughout the review	YES
Selection process	11b	State the process that will be used for selecting studies (such as two independent reviewers) through each phase of the review (that is, screening, eligibility and inclusion in meta-analysis)	YES
Data collection process	11c	Describe planned method of extracting data from reports (such as piloting forms, done independently, in duplicate), any processes for obtaining and confirming data from investigators	YES
Data items	12	List and define all variables for which data will be sought (such as PICO items, funding sources), any pre-planned data assumptions and simplifications	YES
Outcomes and prioritization	13	List and define all outcomes for which data will be sought, including prioritization of main and additional outcomes, with rationale	N/A
Risk of bias in individual studies	14	Describe anticipated methods for assessing risk of bias of individual studies, including whether this will be done at the outcome or study level, or both; state how this information will be used in data synthesis	N/A
Data synthesis	15a	Describe criteria under which study data will be quantitatively synthesised	N/A
	15b	If data are appropriate for quantitative synthesis, describe planned summary measures, methods of handling data and methods of combining data from studies, including any planned exploration of consistency (such as I^2, Kendall's τ)	N/A
	15c	Describe any proposed additional analyses (such as sensitivity or subgroup analyses, meta-regression)	N/A
	15d	If quantitative synthesis is not appropriate, describe the type of summary planned	YES
Meta-bias(es)	16	Specify any planned assessment of meta-bias(es) (such as publication bias across studies, selective reporting within studies)	N/A
Confidence in cumulative evidence	17	Describe how the strength of the body of evidence will be assessed (such as GRADE)	N/A

From: Shamseer L, Moher D, Clarke M, Ghersi D, Liberati A, Petticrew M, Shekelle P, Stewart L, PRISMA-P Group. Preferred reporting items for systematic review and meta-analysis protocols (PRISMA-P) 2015: elaboration and explanation. BMJ. 2015 Jan 2;349(jan02 1):g7647.

For peer review only

Supplementary File 2: Search strategy for Pubmed using the E-utilities

The search on Pubmed was done by running the E-utilities software (1) from NCBI in a shell script (see below “retrieve_guidelines_pubmed.sh”) developed specifically for this project. The combination of the search results into excel tables was done with the R software and two excel related R-packages (see below “CVD_guidelines_search.R”) (2,3,4)

The keyword strings used in this search were:

“CVD cardiovascular diseases”
“HF heart failure”
“CAD coronary artery disease”
“CVA cerebrovascular accident stroke”
“PAD peripheral artery disease”
“HT hypertension”

Pubmed search term per keyword string:

(((guideline[Title]) OR guideline[Publication Type]) AND (keywordString)) AND (("2012"[Date - Publication] : "2023/01/05"Date - Publication]))

Example: For the keyword string “CAD coronary artery disease “, The search query would be:

(((guideline[Title]) OR (guideline[Publication Type])) AND (CAD coronary artery disease)) AND (("2012"[Date - Publication] : "2023/01/05"[Date - Publication]))

Esearch command for each keyword string:

esearch -db pubmed -query "((guideline[Title]) OR guideline[Publication Type]) AND ($KeywordString)" -datetype PDAT -mindate 2012 -maxdate 2023/01/05

This is the equivalent of the manual pubmed search automatized in a bash code (mac-zsh). Only pmids are retrieved using efetch and the parameter: “-format uid”.
Pmids are then sorted for uniqueness using bash. The code then loops over all unique pmids to store publication details into tab-delimited text files using efetch and xtract commands.

Find the original codes attached to this document:

1. *“retrieve_guidelines_pubmed.sh”*
2. *“CVD_guidelines_search.R”*

Code 1: retrieve_guidelines_pubmed.sh

```
#!/bin/zsh

#this code works best on macintosh zsh terminal, to try it on another
shell, replace #!/bin/zsh by #!/bin/sh in the shebang line 1.

#$1 is a text file with keywords strings like "HF heart failure"
#$2 is the start year and $3 is the end year for the search formats:
YYYY or YYYY/MM or YYYY/MM/DD

#lauch code command for 2012-2023/01/05 full search:
#./retrieve_on_keyword_opti.sh keywords.txt 2012 2023/01/05

#the code will create and fill up a set of text and csv files for the
search results.

Lines=$(cat $1|wc -l|sed 's/ //g' |cut -f1) #number of keywords
Date1=$(echo $2 | sed 's/\//_/g'|cut -f1) #reformat dates with
underscores
Date2=$(echo $3 | sed 's/\//_/g'|cut -f1)

PMIDsfile=PMIDs_allkeywords_"$Date1"_to_"$Date2".txt
PMIDKEYSfile=pmid_keys_"$Date1"_to_"$Date2".csv
cat pmid_keys_header.csv > $PMIDKEYSfile

for l in {1..$Lines} #for loop over all keyword strings
do
    Keyword=$(cat $1| head -$l | tail -1)

    echo $l: $Keyword
    if [ -f "$Keyword"_"$Date1"_to_"$Date2"_pmids.txt ];
    then
        rm "$Keyword"_"$Date1"_to_"$Date2"_pmids.txt
    fi
    #from the initial esearch, only pmids are stored into a text file
    esearch -db pubmed -query "((guideline[Title]) OR
guideline[Publication Type]) AND ($Keyword)" -datetype PDAT -mindate $2
-maxdate $3| efetch -format uid
>>"$Keyword"_"$Date1"_to_"$Date2"_pmids.txt

    cat "$Keyword"_"$Date1"_to_"$Date2"_pmids.txt >>tmp1.txt

    for k in $(cat "$Keyword"_"$Date1"_to_"$Date2"_pmids.txt)
    do
        echo "$k,$Keyword" >> tmp_pmid_keys.csv
    done
done

#pmids are now sorted for uniqueness
cat tmp1.txt |cut -f1 | sort -dhu | awk 'NR>1'>tmp2.txt
cat tmp_pmid_keys.csv| sort -k1,1 -u | awk 'NR>1'>>$PMIDKEYSfile
cat tmp2.txt | sort -dhu | awk 'NR>1'>$PMIDsfile
rm tmp1.txt
rm tmp2.txt
rm tmp_pmid_keys.csv

#step2
```

For peer review only

```
Detailsfile=Details_Publications_"$Date1"_to_"$Date2".txt
DOIsfile=DOIs_Publications_"$Date1"_to_"$Date2".txt
Abstractsfile=Abstracts_Publications_"$Date1"_to_"$Date2".txt
Typefile=Pub_types_"$Date1"_to_"$Date2".txt

cat header_opti.txt >$Detailsfile
cat DOIheader.txt >$DOIsfile
cat Typeheader.txt >$Typefile
cat abstractsheader.txt >$Abstractsfile
pLines=$(cat $PMIDsfile|wc -l|sed 's/ //g' |cut -f1)

echo "running for $pLines entries"

for p in {1..$pLines} #for loop over all unique pmid to store
publication details into tab delimited text files
do
    PMID=$(cat $PMIDsfile| head -$p | tail -1)

    if [ $(expr $p % 50) -eq 0 ]
    then
        echo "$p over $pLines"
    fi
    #efetch efficiency depends a lot on internet connexion speed and
stability
     efetch -db pubmed -id $PMID -format xml > temp.xml
    cat temp.xml| xtract -pattern PubmedArticle -def "-" -element
MedlineCitation/PMID PubDate/Year PubDate/Month PubDate/Day
PubDate/MedlineDate DateRevised/Month Article/ArticleTitle
Article/VernacularTitle Journal/Title JournalIssue/Volume
JournalIssue/Issue Pagination/MedlinePgn Abstract
MedlineJournalInfo/Country -block Author -tab ", "  -sep " " -element
LastName,Initials >> $Detailsfile

    cat temp.xml | xtract -pattern PubmedArticle -def "-" -element
MedlineCitation/PMID -block Article -tab "; "  -sep "; " -element
ELocationID@EIdType ELocationID >>$DOIsfile

    cat temp.xml | xtract -pattern PubmedArticle -def "-" -element
MedlineCitation/PMID -block Article -tab "; "  -sep "; " -element
PublicationTypeList/PublicationType >>$Typefile

     echo "$(cat temp.xml | xtract -pattern PubmedArticle -def "-" -
element MedlineCitation/PMID -block Article -tab "; "  -sep "; " -def
"-"  -element Abstract/AbstractText | tr -d '\n')\n" >>$Abstractsfile
done

echo "its over";
rm temp.xml
```

Code 2: CVD_guidelines_search.R

```
#You must first run the shell script "retrieve_guidelines_pubmed.sh"

library(xlsx)
library(readxl)

fileversion<-"_v06"
period<-"2012_to_2023/05/01"
filesLocation<-"~/Documents/oneshots/Litterature_search/"

pubmed<-
read.delim(paste0(filesLocation,"Details_Publications_",period,".txt"))
dois<-
read.delim(paste0(filesLocation,"DOIs_Publications_",period,".txt"))
abstracts<-
read.delim(paste0(filesLocation,"Abstracts_Publications_",period,".txt"
))
keys<-read.csv(paste0(filesLocation,"pmid_keys_",period,".csv"))
types<-read.delim(paste0(filesLocation,"Pub_types_",period,".txt"))

getDOI<-function(Elo){
  a<-paste0(strsplit(Elo,split="; ")[[1]],"")

  if (length(a)==2){
    return(paste0(a[2][a[1]=="doi"][1],""))
  }
  if (length(a)>2){
    b<-matrix(a,ncol=2)
    return(paste0(b[,2][b[,1]=="doi"][1],""))
  }
  else{
    return ("")}
}

dois$DOI<-sapply(dois$ELocationIDs, function(x) getDOI(x))
dois$DOI[dois$DOI!=""]<-paste0("http://doi.org/",dois$DOI[dois$DOI!=""
&!is.na(dois$DOI)])

abstracts$depression<-sapply(abstracts$Abstract,function(x)
grepl("depress",x,ignore.case = T))
abstracts$psych<-sapply(abstracts$Abstract,function(x)
grepl("psych",x,ignore.case = T))
abstracts$mental<-sapply(abstracts$Abstract,function(x) grepl(" mental
",x,ignore.case = T)|grepl(" mentally ",x,ignore.case = T))

pubmed$keywords<-
sapply(pubmed$PMID,function(x)paste(keys$Keyword[keys$PMID==x],collapse
=", "))

if(identical(pubmed$PMID,dois$PMID)){
  pubmed$DOI<-dois$DOI
  pubmed$DOI[pubmed$DOI=="http://doi.org/NA"]<-""
}
if(identical(pubmed$PMID,abstracts$PMID)){
  pubmed$depression<-abstracts$depression
  pubmed$psych<-abstracts$psych
  pubmed$mental<-abstracts$mental
```

For peer review only

```
  pubmed$Abstract[abstracts$Abstract!=""]<-"accessible"
}
if(identical(pubmed$PMID,types$PMID)){
  pubmed$Publication_types<-types$Publication_types
}

pubmed[,"Relev/NR = 1/0 ?"]<-character(nrow(pubmed))
pubmed<-pubmed[,c(1:6,ncol(pubmed),7:(ncol(pubmed)-1))]

write.xlsx(pubmed,paste0(filesLocation,"litterature_on_CVD_and_MH_Every
thing_",period,fileversion,".xlsx"),sheetName =
paste0("Pubmed_",period),showNA = F,row.names = F)
write.xlsx(pubmed[pubmed$depression|pubmed$psych|pubmed$mental,],paste0
(filesLocation,"litterature_on_CVD_and_MH_Everything_",period,fileversi
on,".xlsx"),sheetName = paste0("Pubmed_PSY",period),showNA =
F,row.names = F,append = T)

selection<-c("PMID",    "Year",     "Relev/NR = 1/0 ?",     "Title",
      "Journal",  "DOI",      "Publication_types")

write.xlsx(pubmed[,selection][grepl("guideline",pubmed$Publication_type
s, ignore.case = T) & pubmed$VernacularTitle=="-
",],paste0(filesLocation,"litterature_on_CVD_trimed_",period,fileversio
n,".xlsx"),sheetName = paste0("Guidelines_PubType_ENG"),showNA =
F,row.names = F)
write.xlsx(pubmed[,selection][!grepl("guideline",pubmed$Publication_typ
es, ignore.case = T) & pubmed$VernacularTitle=="-
",],paste0(filesLocation,"litterature_on_CVD_trimed_",period,fileversio
n,".xlsx"),sheetName = paste0("Guidelines_Title_ENG"),showNA =
F,row.names = F,append=T)
write.xlsx(pubmed[,selection][pubmed$VernacularTitle!="-
",],paste0(filesLocation,"litterature_on_CVD_trimed_",period,fileversio
n,".xlsx"),sheetName = paste0("non_English_pubs",showNA = F,row.names =
F,append=T)
```

For peer review only

References

1. Sayers E. The E-utilities In-Depth: Parameters, Syntax and More. 2009 May 29 [Updated 2022 Nov 30]. In: Entrez Programming Utilities Help [Internet]. Bethesda (MD): National Center for Biotechnology Information (US); 2010-. Available from: https://www.ncbi.nlm.nih.gov/books/NBK25499/

2. R Core Team (2022). R: A language and environment for statistical computing. R Foundation for Statistical Computing, Vienna, Austria. URL https://www.R-project.org/

3. Dragulescu A, Arendt C (2020). _xlsx: Read, Write, Format Excel 2007 and Excel 97/2000/XP/2003 Files_. R package version 0.6.5, https://CRAN.R-project.org/package=xlsx

4. Wickham H, Bryan J (2022). _readxl: Read Excel Files_. R package version 1.4.1, https://CRAN.R-project.org/package=readxl

For peer review only

Clinical practice guidelines for cardiovascular disease: how is depression addressed? Protocol for a systematic review

Journal:	*BMJ Open*
Manuscript ID	bmjopen-2023-071940.R1
Article Type:	Protocol
Date Submitted by the Author:	24-Mar-2023
Complete List of Authors:	Blatch Armon, Dana; International Center for Multimorbidity and Complexity in Medicine, University of Zurich, University Hospital Basel (Department of Psychosomatic Medicine), Merian Iselin Klinik, Basel, Switzerland<br>Buhayer, Aliki; International Center for Multimorbidity and Complexity in Medicine, University of Zurich, University Hospital Basel (Department of Psychosomatic Medicine), Merian Iselin Klinik, Basel, Switzerland<br>Dobretz, Kevin; Geneva University Hospitals, Cardiology<br>Meinlschmidt, Gunther; University Hospital Basel and University of Basel, Department of Psychosomatic Medicine; Cognitive Behavioural Therapy International Psychoanalytic University<br>Battegay, Edouard; International Center for Multimorbidity and Complexity in Medicine, University of Zurich, University Hospital Basel (Department of Psychosomatic Medicine), Merian Iselin Klinik, Basel, Switzerland; University Hospital Basel, Psychosomatic Medicine
**Primary Subject Heading**:	Cardiovascular medicine
Secondary Subject Heading:	Evidence based practice, General practice / Family practice, Mental health, Patient-centred medicine
Keywords:	CARDIOLOGY, Depression & mood disorders < PSYCHIATRY, MENTAL HEALTH, PRIMARY CARE

SCHOLARONE™
Manuscripts

Clinical practice guidelines for cardiovascular disease: how is depression addressed? Protocol for a systematic review

Blatch Armon Dana*[1], Buhayer Aliki*[1], Dobretz Kevin[2], Meinlschmidt Gunther[3,4], Battegay Edouard[1,5]

Correspondence to: Dr Dana Blatch Armon; dana.blatch@mail.huji.ac.il
Merian Iselin Klinik, Föhrenstrasse 2, 4009 Basel, Switzerland

Author affiliations

* Contributed equally to this paper
1 International Center for Multimorbidity and Complexity in Medicine, University of Zurich, University Hospital Basel (Department of Psychosomatic Medicine), Merian Iselin Klinik, Basel, Switzerland
2 Geneva University Hospitals, Department of Cardiology, Geneve, Switzerland
3 Department of Psychosomatic Medicine, University Hospital Basel and University of Basel, Basel, Switzerland
4 Division of Clinical Psychology and Cognitive Behavioural Therapy, International Psychoanalytic University (IPU) Berlin, Berlin, Germany
5 University Hospital Basel, Department of Psychosomatic Medicine, Basel, Switzerland

Emails of all authors
dana.blatch@mail.huji.ac.il
ab@prismscientific.ch
Kevin.Dobretz@hcuge.ch
gunther.meinlschmidt@unibas.ch
edouard.battegay@uzh.ch

Orcid authors:
Dana https://orcid.org/0000-0002-2160-9144
Aliki https://orcid.org/0000-0002-6538-0273
Edouard https://orcid.org/0000-0001-9202-5034
Gunther https://orcid.org/0000-0002-3488-193X

Word count: 1820

For peer review only

For peer review only - http://bmjopen.bmj.com/site/about/guidelines.xhtml

Abstract

Introduction

Depression frequently affects patients with cardiovascular disease (CVD). When these conditions co-occur, outcomes such as quality of life and life expectancy worsen. In everyday practice, this specific and prevalent disease-disease interaction complicates patient management. Clinical practice guidelines (CPG) aim to provide the best available advice for clinical decision-making to improve patient care. This study will aim to evaluate how CPGs specifically address depression in patients with CVD, and whether they provide any operational guidance for screening and management of depression in the primary care and outpatient setting.

Methods and analysis

We will conduct a systematic review of clinical practice guidelines on CVD management published from 2012 to 2023. A broad literature search for guidelines will be performed through electronic medical databases, grey literature search tools, and websites of national and professional medical organizations.
Based on the inclusion criteria, two independent reviewers will evaluate eligible guidelines for screening and management recommendations on depression in patients with CVD. Additional points to be evaluated will be any mention of drug-drug or drug-disease interactions, other aspects of specific relevance to treating physicians, as well as general information on mental health. We will assess the quality of CPGs with a recommendation regarding depression in CVD patients using the Appraisal of Guidelines for Research and Evaluation II (AGREE-II).

Ethics and dissemination

As this systematic review is based on available published data, ethics approval and consent are not applicable. Our intent is that our results will be published in a peer-reviewed journal, presented at international scientific meetings, and distributed to healthcare providers.

PROSPERO registration number CRD42022384152

Article summary

Strengths and limitations of this study

- The rigorous literature search method we developed aims to produce an extensive record of CVD CPGs, to accurately describe how depression screening and management is addressed in these patients.

For peer review only - http://bmjopen.bmj.com/site/about/guidelines.xhtml

- Our literature search will cover the past 10 years to provide insights into current and clinically relevant guidelines, while also facilitating the identification of possible trends in recommendations.
- This systematic review will use the Appraisal of Guidelines for Research & Evaluation (AGREE) II instrument to evaluate the quality of the selected guidelines, including all relevant recommendations.
- This study will include only CPGs published in English, excluding any guidelines produced in other languages.

Introduction

Despite substantial advances in the field, cardiovascular disease (CVD) is still the leading cause of morbidity and mortality in the world (1). Improvements in primary prevention and management of risk factors have lowered the risk of cardiovascular events and reduced age-specific mortality over the past decades (1,2). However, higher life expectancy has led to a rise in comorbid conditions in CVD patients (3). Multimorbidity, commonly described as the occurrence of two or more chronic conditions in one individual (4), is associated with increased mortality, decreased quality of life, and increased use of healthcare services (3,5,6), including visits to general practitioners and other medical specialists (7). In addition, multimorbidity leads to challenging situations in the treatment and management of these patients, further complicating medical decision-making (7,8). The relationship between CVD and multimorbidity is well established (9–11), and among multimorbidities, depression is one of the most common co-occurring conditions (12).

Depression is a common mental disorder that interferes with a person's mood and behavior (13). It affects up to 10% of adults throughout their lifetime (14) and is considered to be the leading cause of disability worldwide (15). In patients with CVD, the occurrence of depression is estimated to be 1 in 5 (12). When controlling for traditional CVD risk factors, the presence of depressive symptoms even without a clinical diagnosis of depressive disorder can predict the incidence of coronary artery disease in healthy individuals (16), as well as secondary events in these patients (17–20), and adverse outcomes following coronary artery bypass grafting (21). In patients with CVD, depression was also associated with an elevated risk of failure to adhere to medications and a healthy lifestyle (22,23). Patients with depression may be less likely to engage in behaviors that lower the risk of future cardiovascular events, such as physical activity, dietary modifications, smoking cessation, stress management, and treatments against substance abuse (24,25).

Inadequate treatment of depression is associated with a higher risk of adverse cardiovascular events (26). Primary care physicians play an important role in recognizing and managing depression since an estimated 60% of mental health care delivery occurs in the primary care setting (27). Despite high evidence on the importance of diagnosis and treatment of depression (28), implementation in practice remains limited (29), and over 50% of patients with depression in primary care remain unrecognized and inadequately treated (30–33).

Clinical practice guidelines (CPG) provide the best available evidence to support clinical decision-making and aim to improve care and outcomes (34), however, they vary in their recommendations regarding screening for depression. For example, the US Preventive Services

For peer review only

For peer review only - http://bmjopen.bmj.com/site/about/guidelines.xhtml

Task Force (USPSTF) recommends screening for depression in the general adult population (35), whereas official guidelines by the National Institute for Health and Clinical Excellence (NICE), as well as The Canadian Task Force on Preventive Health Care (CTFPHC) recommend not to screen routinely for depression in the primary care setting (36,37).
A recent state-of-the-art review on depression in patients with CVD showed that guideline recommendations for screening for depression are only partially available and inconsistent (38). The recommendations tended to focus on specific sub-patient groups and lacked a systematic approach, also regarding which screening tools to use, leaving an incomplete picture of practical recommendations for practitioners to follow in their daily practice (38).

Evidence gap

The systematic review of existing CPGs is an approach that is increasingly being used in an attempt to characterize the nature of clinical guidance on a topic of interest (34). To our knowledge, no such review has yet been performed regarding how depression is addressed in CVD guidelines. As standardized screening and treatment pathways offer the potential for early identification and improved management of both CVD and depression in patients treated for specific CV diseases (39–41), we aim to conduct such a review in order to provide a clear picture of the recommendations currently available and identify gaps in knowledge. This step is crucial to provide guidance for future research and, ultimately, for improving the quality of evidence-based clinical recommendations.

Aim

The aim of this systematic review will be to identify, appraise and describe how CPGs for CVD management specifically address depression, and whether they provide operational guidance for screening and management to practitioners.
This systematic review is part of a larger review currently in process that is meant to provide a comprehensive picture of knowledge and evidence gathered so far on patients with concurrent CVD and depression. It is also intended to identify knowledge gaps in the current state of clinical guidance, thereby promoting further research to produce evidence-based guidance for future guidelines to further improve treatment for patients.

Objectives

1. Identify published CPGs on the management of CVD in the primary care and outpatient setting.
2. Among all qualifying CPGs on CVD, identify those that mention and provide recommendations for depression as comorbidity or multimorbidity.
3. Assess the quality of recommendations using the Appraisal of Guidelines for Research and Evaluation version 2 (AGREE II) instrument.
4. Summarize the current situation with regard to the consideration of depression in CPG on CVD.

For peer review only - http://bmjopen.bmj.com/site/about/guidelines.xhtml

Methods and analysis

For the preparation of this protocol we followed specific methodological guidance for conducting systematic reviews of CPGs, as such reviews require a tailored approach to design and execution compared with other systematic reviews (34). This protocol follows the PRISMA-P guidelines for the reporting of systematic review protocols (42). The completed checklist for this protocol is available in the Supplementary File 1.

Patient and public involvement

Patients and/or the public were not involved in the design, or conduct, or reporting, or dissemination plans of this research protocol.

Eligibility criteria

The PICAR (43) framework was used to guide inclusion and exclusion criteria and is described in Table 1, as recommended by the PRISMA P framework for protocols of systematic reviews. These selected guidelines will be assessed for recommendations regarding depression in patients with CVD. We will use the Preferred Reporting Items for Systematic Reviews and Meta-Analyses guidelines (PRISMA) to report the results of this review (44).

Table 1 PICAR statement

P: Population, clinical indication(s) and condition(s)	Study population: adults 18 and above with CVD<br>Clinical indication: Management of patients with CVD in the primary/specialty outpatient setting<br>Clinical condition: CVD
I: Intervention(s)	Any intervention focusing on diagnosis and/or treatment of depression
C: Comparator(s)	No comparator. All aspects of depression diagnosis and management will be taken into consideration.
A: Attributes of eligible CPGs	• Language: English<br>• Time range: 10 years, starting from search date. CPGs that were published before 2013 and have not been renewed or updated since are considered out of date on the basis of the rapid advances in the field of CVD over the past decade.<br>• Publishing region: no restriction<br>• Versions: latest versions only<br>• Developing publishing organization: endorsed by national or international scientific societies, professional colleges, charitable organizations, and government organizations will be included.<br>• Publication status: only published CPGs.<br>• Development process: explicitly evidence- and/or consensus-

For peer review only

	based<br>● System of rating evidence: Use of a system to rate the level of evidence within CPGs is not an eligibility criterion; but will be taken into consideration when available and stated clearly<br>● Scope: CPGs for the general management of adult patients with CVD (including heart failure, coronary artery disease, stroke, peripheral artery disease, aortic disease, diabetes, dyslipidemia and hypertension management) in the community setting.
R: Recommendations characteristics	Recommendations covering screening, diagnosis, and pharmacological and non-pharmacological management of depression are of interest.

CVD, cardiovascular disease; CPG, clinical practice guidelines.

For peer review only

Search strategy and information sources

To identify all relevant CPGs, the search strategy was developed on the basis of the Johnston paper recommendations for a systematic review of CPGs and under the guidance of an experienced bioinformatician (KD). We will conduct a systematic search in the following databases: PUBMED, TRIP, EMBASE, Guidelines international network (GIN), and Guideline central, using search terms and methods adjusted to the specificities of each database (example in Supplementary file 2). In order to locate additional relevant CPGs not available in academic journals, we will extend our search range to include additional grey literature that will target guidelines by specifically searching on the Society guideline link on Uptodate, World Health Organization guidelines section, and the websites of national and professional medical organizations (e.g., National Institute for Health and Care Excellence (NICE), Scottish Intercollegiate Guidelines Network (SIGN)).

Guideline selection

All results collected in the literature search will be summarized in a table and submitted to two independent reviewers (DB and AB) who will conduct a two-step selection procedure for eligibility, based on the pre-defined inclusion and exclusion criteria:

1. First step - Titles and abstracts will be screened for relevance. Duplicates will be removed in this step. Potentially relevant CPGs where an abstract is not provided will be passed to the second screening stage.
2. Second step - full-text screening to assess CPGs for final inclusion in the review.

Records will be excluded if both reviewers agreed they were not eligible. Any disagreements will be resolved by consensus. If consensus cannot be met between the two reviewers, a third reviewer (EB) will also assess the particular record to determine its eligibility.

For peer review only - http://bmjopen.bmj.com/site/about/guidelines.xhtml

Inclusion criteria

1. CPGs for general outpatient management of adult patients with CVD (including specific guidelines for heart failure, coronary artery disease, stroke, peripheral arterial disease, aortic disease, diabetes, dyslipidemia, and hypertension management) in the community setting.
2. The CPG is published in English.
3. The CPG was published within the past 10 years.
4. The CPG is the most recent version.
5. The full CPG is available online.
6. The CPG was written by national and international academic organizations.

Exclusion criteria

1. Guidelines limited to a specific aspect of CVD management, such as screening, pharmacological management, specific interventions, acute and hospital setting, or not relevant to primary care or outpatient care.
2. Target population under 18 years of age.

Data extraction and analysis

Data management and extraction

Once the final set of included CPGs has been obtained, DB and AB will conduct a search in the full text for any mention of depression, using the search terms “depress” “mental” “psych” and “mood”. These search terms will be used to identify all the places within the guidelines which mention the topic of mental health, in order to extract any information specifically regarding depression. Additional information regarding other mental disorders will only be evaluated for relevance and briefly summarized and presented, as it is not the focus of this work.
First, all results collected will be summarized in a table containing the CPG title, year of publication, organization and country, and the exact context in the document (page number, chapter, line, table, or figure number).
Second, DB and AB will assess and categorize each result for any operational guidance on screening and/or management of depression in patients with CVD. Any mention of interactions and points of consideration for treating physicians, as well as general information on mental health issues will also be included.
Specifically, the following aspects will be examined and classified:

- Screening recommendation: population, how to screen (who, what screening tool), when (and how often), and what to do with a positive screening result (treatment, referral).
- Management – pharmacological and non-pharmacological treatment modalities, drug-drug or drug-disease interactions to consider.

Special consideration will be given to the level of evidence on which the recommendation is based, and will be reported when available. In addition, the quality of the selected CPGs will be assessed using the updated AGREE-II instrument (45). As the study progresses, the datasheet

For peer review only

may evolve to fit the study's objectives better. The full list of variables of interest is shown in Table 2.

Table 2 List of variables

Name of variable
CPG topic (CVD or specific CV-related conditions, including CAD, HF, stroke, PAD, aortic disease, hypertension, diabetes, dyslipidemia)
CPG title
Organization/society responsible for the guideline
Year of publication
Source of funding
Country
Grading system for recommendations
Depression, any mention
Depression screening and management, any recommendation
CVD and depression interactions (drug-drug, disease-drug)
Level of evidence of depression recommendation
Overall AGREE-II score of CPG

CPG, clinical practice guideline; CV, cardiovascular; CVD, cardiovascular disease; HF, heart failure; CAD, coronary artery disease; PAD, peripheral artery disease.
AGREE-II, Appraisal of Guidelines for Research and Evaluation II.

Results

The systematic review will aim at providing results for the following planned steps:

1. A flowchart of the search strategy.
2. A detailed summary of available recommendations (any type) on depression listed by identified CPG on CVD.
3. Quality assessment sheets of CPGs using the AGREE-II methodology.

Significance of the study

Patients with CVD have a worse prognosis if they suffer from depression. This study aims to establish what guidance CPGs for CVD provide regarding screening and management of depression. By evaluating the current state of recommendations available and identifying the gaps in knowledge, this study may serve as a guide for tailoring future research in this field. This

For peer review only - http://bmjopen.bmj.com/site/about/guidelines.xhtml

step is crucial for the development of evidence-based recommendations for future CPGs aiming at providing better guidance on the management of depression in patients with CVD.

Footnotes

Ethics and dissemination

As this study is a systematic review, a secondary analysis of published data will be performed, and no human participants involved in this research. Therefore, in this situation ethics approval and consent are not applicable.

This study is registered on the International Prospective Register of Systematic Reviews (PROSPERO) as of 26 December 2022 (registration number: CRD42022384152) and will be updated as needed and as main results will be published.

The results of this study will be reported in accordance with the PRISMA statement (44) and disseminated through publication in a peer-reviewed journal, presentation at international scientific conferences, and contact with medical associations responsible for writing guidelines.

Author contributions

EB, AB and DB were involved in the conceptualization of the study. EB, AB, DB and GM designed the protocol and developed the methodology for the literature search strategy. KD will conduct the literature search. DB and AB will conduct the data analysis, quality assessment, and formal analysis. EB will supervise the study and is in charge of Funding acquisition. All authors have made important intellectual content contributions to the protocol, and reviewed and edited its final version.

Funding

None declared for AB and DB and KD.
EB received a nonrestricted educational and research grant of the Zurich Academy of Internal Medicine, https://www.my-zaim.ch.
GM received funding from the Stanley Thomas Johnson Stiftung & Gottfried und Julia Bangerter-Rhyner-Stiftung under projects no. PC 28/17 and PC 05/18, from Gesundheitsförderung Schweiz under project no. 18.191/K50001, from the Swiss Heart Foundation under project no. FF21101, from the Research Foundation of the International Psychoanalytic University (IPU) Berlin, from the Swiss National Science Foundation (SNSF) under project no. 100014_135328, in the context of a Horizon Europe project from the Swiss State Secretariat for Education, Research and Innovation (SERI) under contract number 22.00094, and from Wings Health Inc. in the context of a proof-of-concept study. GM is co-

For peer review only - http://bmjopen.bmj.com/site/about/guidelines.xhtml

founder, member of the board, and shareholder of Therayou AG, which is active in the field of digital and blended mental healthcare.

Competing interests

None declared.

References

1. Capewell S, O'Flaherty M. Rapid mortality falls after risk-factor changes in populations. Lancet Lond Engl. 2011 Aug 27;378(9793):752–3.

2. O'Flaherty M, Buchan I, Capewell S. Contributions of treatment and lifestyle to declining CVD mortality: why have CVD mortality rates declined so much since the 1960s? Heart Br Card Soc. 2013 Feb;99(3):159–62.

3. van den Akker M, Buntinx F, Metsemakers JF, Roos S, Knottnerus JA. Multimorbidity in general practice: prevalence, incidence, and determinants of co-occurring chronic and recurrent diseases. J Clin Epidemiol. 1998 May;51(5):367–75.

4. Harrison C, Fortin M, van den Akker M, Mair F, Calderon-Larranaga A, Boland F, et al. Comorbidity versus multimorbidity: Why it matters. J Multimorb Comorbidity [Internet]. 2021 Jan 1 [cited 2022 Oct 14];11:263355652199399. Available from: http://journals.sagepub.com/doi/10.1177/2633556521993993

5. Pati S, Swain S, Hussain MA, van den Akker M, Metsemakers J, Knottnerus JA, et al. Prevalence and outcomes of multimorbidity in South Asia: a systematic review. BMJ Open. 2015 Oct 7;5(10):e007235.

6. Salisbury C, Johnson L, Purdy S, Valderas JM, Montgomery AA. Epidemiology and impact of multimorbidity in primary care: a retrospective cohort study. Br J Gen Pract [Internet]. 2011 Jan 1 [cited 2022 Oct 17];61(582):e12–21. Available from: https://bjgp.org/lookup/doi/10.3399/bjgp11X548929

7. Starfield B, Lemke KW, Bernhardt T, Foldes SS, Forrest CB, Weiner JP. Comorbidity: implications for the importance of primary care in 'case' management. Ann Fam Med. 2003 Jun;1(1):8–14.

8. Farmer C, Fenu E, O'Flynn N, Guthrie B. Clinical assessment and management of multimorbidity: summary of NICE guidance. BMJ. 2016 Sep 21;354:i4843.

9. Aarts S, van den Akker M, Tan FES, Verhey FRJ, Metsemakers JFM, van Boxtel MPJ. Influence of multimorbidity on cognition in a normal aging population: a 12-year follow-up in the Maastricht Aging Study. Int J Geriatr Psychiatry. 2011 Oct;26(10):1046–53.

10. Bang D, Xu J, Keenan R, Pike V, Lehmann R, Tenner C, et al. Cardiovascular Disease Prevalence in Patients with Osteoarthritis, Gout, or Both. Bull Hosp Jt Dis 2013. 2016 Jun;74(2):113–8.

For peer review only - http://bmjopen.bmj.com/site/about/guidelines.xhtml

11. Zander N, Schäfer I, Radtke M, Jacobi A, Heigel H, Augustin M. Dermatological comorbidity in psoriasis: results from a large-scale cohort of employees. Arch Dermatol Res. 2017 Jul;309(5):349–56.

12. Thombs BD, Bass EB, Ford DE, Stewart KJ, Tsilidis KK, Patel U, et al. Prevalence of depression in survivors of acute myocardial infarction: Review of the evidence. J Gen Intern Med [Internet]. 2006 Jan [cited 2022 Sep 7];21(1):30–8. Available from: http://link.springer.com/10.1111/j.1525-1497.2005.00269.x

13. American Psychiatric Association. Diagnostic and Statistical Manual of Mental Disorders [Internet]. Fifth Edition. American Psychiatric Association; 2013 [cited 2022 Dec 27]. Available from: https://psychiatryonline.org/doi/book/10.1176/appi.books.9780890425596

14. Lim GY, Tam WW, Lu Y, Ho CS, Zhang MW, Ho RC. Prevalence of Depression in the Community from 30 Countries between 1994 and 2014. Sci Rep. 2018 Feb 12;8(1):2861.

15. World Health Organization. Depression and other common mental disorders: global health estimates [Internet]. Geneva: World Health Organization; 2017. Available from: https://apps.who.int/iris/handle/10665/254610

16. Lett HS, Blumenthal JA, Babyak MA, Sherwood A, Strauman T, Robins C, et al. Depression as a risk factor for coronary artery disease: evidence, mechanisms, and treatment. Psychosom Med. 2004 Jun;66(3):305–15.

17. Stewart R. Depression and cardiovascular morbidity and mortality: cause or consequence? Eur Heart J [Internet]. 2003 Nov [cited 2022 Dec 27];24(22):2027–37. Available from: https://academic.oup.com/eurheartj/article-lookup/doi/10.1016/j.ehj.2003.08.017

18. Barth J, Schumacher M, Herrmann-Lingen C. Depression as a risk factor for mortality in patients with coronary heart disease: a meta-analysis. Psychosom Med. 2004 Dec;66(6):802–13.

19. van Melle JP, de Jonge P, Spijkerman TA, Tijssen JGP, Ormel J, van Veldhuisen DJ, et al. Prognostic association of depression following myocardial infarction with mortality and cardiovascular events: a meta-analysis. Psychosom Med. 2004 Dec;66(6):814–22.

20. Evans DL, Charney DS, Lewis L, Golden RN, Gorman JM, Krishnan KRR, et al. Mood disorders in the medically ill: scientific review and recommendations. Biol Psychiatry. 2005 Aug 1;58(3):175–89.

21. Borowicz LJ, Royall R, Grega M, Selnes O, Lyketsos C, McKhann G. Depression and cardiac morbidity 5 years after coronary artery bypass surgery. Psychosomatics. 2002 Dec;43(6):464–71.

22. Gehi A, Haas D, Pipkin S, Whooley MA. Depression and medication adherence in outpatients with coronary heart disease: findings from the Heart and Soul Study. Arch Intern Med. 2005 Nov 28;165(21):2508–13.

For peer review only

For peer review only - http://bmjopen.bmj.com/site/about/guidelines.xhtml

23. Sin NL, Kumar AD, Gehi AK, Whooley MA. Direction of Association Between Depressive Symptoms and Lifestyle Behaviors in Patients with Coronary Heart Disease: the Heart and Soul Study. Ann Behav Med Publ Soc Behav Med. 2016 Aug;50(4):523–32.

24. Tang HYJ, Sayers SL, Weissinger G, Riegel B. The role of depression in medication adherence among heart failure patients. Clin Nurs Res. 2014 Jun;23(3):231–44.

25. Stapelberg NJC, Neumann DL, Shum DHK, McConnell H, Hamilton-Craig I. A topographical map of the causal network of mechanisms underlying the relationship between major depressive disorder and coronary heart disease. Aust N Z J Psychiatry. 2011 May;45(5):351–69.

26. Bangalore S, Shah R, Pappadopulos E, Deshpande CG, Shelbaya A, Prieto R, et al. Cardiovascular hazards of insufficient treatment of depression among patients with known cardiovascular disease: a propensity score adjusted analysis. Eur Heart J Qual Care Clin Outcomes. 2018 Oct 1;4(4):258–66.

27. Frank RG, Huskamp HA, Pincus HA. Aligning incentives in the treatment of depression in primary care with evidence-based practice. Psychiatr Serv Wash DC. 2003 May;54(5):682–7.

28. Bhattacharjee S, Goldstone L, Vadiei N, Lee JK, Burke WJ. Depression Screening Patterns, Predictors, and Trends Among Adults Without a Depression Diagnosis in Ambulatory Settings in the United States. Psychiatr Serv Wash DC. 2018 Oct 1;69(10):1098–100.

29. Fuster V, Kelly BB, Vedanthan R. Global cardiovascular health: urgent need for an intersectoral approach. J Am Coll Cardiol. 2011 Sep 13;58(12):1208–10.

30. Smithson S, Pignone MP. Screening Adults for Depression in Primary Care. Med Clin North Am [Internet]. 2017 Jul [cited 2022 Sep 30];101(4):807–21. Available from: https://linkinghub.elsevier.com/retrieve/pii/S0025712517300330

31. Trivedi MH, Jha MK, Kahalnik F, Pipes R, Levinson S, Lawson T, et al. VitalSign(6): A Primary Care First (PCP-First) Model for Universal Screening and Measurement-Based Care for Depression. Pharm Basel Switz. 2019 May 14;12(2).

32. Olfson M, Blanco C, Marcus SC. Treatment of Adult Depression in the United States. JAMA Intern Med. 2016 Oct 1;176(10):1482–91.

33. Kato E, Borsky AE, Zuvekas SH, Soni A, Ngo-Metzger Q. Missed Opportunities for Depression Screening and Treatment in the United States. J Am Board Fam Med JABFM. 2018 Jun;31(3):389–97.

34. Johnston A, Kelly SE, Hsieh SC, Skidmore B, Wells GA. Systematic reviews of clinical practice guidelines: a methodological guide. J Clin Epidemiol [Internet]. 2019 Apr [cited 2022 Dec 27];108:64–76. Available from: https://linkinghub.elsevier.com/retrieve/pii/S0895435618302877

For peer review only - http://bmjopen.bmj.com/site/about/guidelines.xhtml

35. Siu AL, Bibbins-Domingo K, Grossman DC, Baumann LC, Davidson KW, Ebell M, et al. Screening for Depression in Adults: US Preventive Services Task Force Recommendation Statement. JAMA. 2016 Jan 26;315(4):380–7.

36. National Institute for Health and Care Excellence. Depression in adults: treatment and management [NG222] [Internet]. 2022 [cited 2022 Dec 30]. Available from: https://www.nice.org.uk/guidance/ng222

37. Joffres M, Jaramillo A, Dickinson J, Lewin G, Pottie K, Shaw E, et al. Recommendations on screening for depression in adults. CMAJ Can Med Assoc J J Assoc Medicale Can. 2013 Jun 11;185(9):775–82.

38. Jha MK, Qamar A, Vaduganathan M, Charney DS, Murrough JW. Screening and Management of Depression in Patients With Cardiovascular Disease. J Am Coll Cardiol [Internet]. 2019 Apr [cited 2022 Sep 27];73(14):1827–45. Available from: https://linkinghub.elsevier.com/retrieve/pii/S0735109719305133

39. Berkman LF, Blumenthal J, Burg M, Carney RM, Catellier D, Cowan MJ, et al. Effects of treating depression and low perceived social support on clinical events after myocardial infarction: the Enhancing Recovery in Coronary Heart Disease Patients (ENRICHD) Randomized Trial. JAMA. 2003 Jun 18;289(23):3106–16.

40. Glassman AH, O'Connor CM, Califf RM, Swedberg K, Schwartz P, Bigger JTJ, et al. Sertraline treatment of major depression in patients with acute MI or unstable angina. JAMA. 2002 Aug 14;288(6):701–9.

41. Kim JM, Stewart R, Lee YS, Lee HJ, Kim MC, Kim JW, et al. Effect of Escitalopram vs Placebo Treatment for Depression on Long-term Cardiac Outcomes in Patients With Acute Coronary Syndrome: A Randomized Clinical Trial. JAMA. 2018 Jul 24;320(4):350–8.

42. PRISMA-P Group, Moher D, Shamseer L, Clarke M, Ghersi D, Liberati A, et al. Preferred reporting items for systematic review and meta-analysis protocols (PRISMA-P) 2015 statement. Syst Rev [Internet]. 2015 Dec [cited 2022 Dec 30];4(1):1. Available from: https://systematicreviewsjournal.biomedcentral.com/articles/10.1186/2046-4053-4-1

43. Methley AM, Campbell S, Chew-Graham C, McNally R, Cheraghi-Sohi S. PICO, PICOS and SPIDER: a comparison study of specificity and sensitivity in three search tools for qualitative systematic reviews. BMC Health Serv Res [Internet]. 2014 Dec [cited 2022 Dec 30];14(1):579. Available from: https://bmchealthservres.biomedcentral.com/articles/10.1186/s12913-014-0579-0

44. Page MJ, McKenzie JE, Bossuyt PM, Boutron I, Hoffmann TC, Mulrow CD, et al. The PRISMA 2020 statement: an updated guideline for reporting systematic reviews. BMJ [Internet]. 2021 Mar 29 [cited 2022 Dec 30];n71. Available from: https://www.bmj.com/lookup/doi/10.1136/bmj.n71

45. Brouwers MC, Kerkvliet K, Spithoff K, AGREE Next Steps Consortium. The AGREE Reporting Checklist: a tool to improve reporting of clinical practice guidelines. BMJ [Internet]. 2016 Mar 8 [cited 2022 Dec 30];i1152. Available from: https://www.bmj.com/lookup/doi/10.1136/bmj.i1152

For peer review only

For peer review only

For peer review only - http://bmjopen.bmj.com/site/about/guidelines.xhtml

Supplementary File 1: Preferred Reporting Items for Systematic Review and Meta-Analysis for Protocols (PRISMA-P) checklist

Section and topic	Item No	Checklist item	Our response
**ADMINISTRATIVE INFORMATION**			
Title:			
Identification	1a	Identify the report as a protocol of a systematic review	YES
Update	1b	If the protocol is for an update of a previous systematic review, identify as such	N/A
Registration	2	If registered, provide the name of the registry (such as PROSPERO) and registration number	YES
Authors:			
Contact	3a	Provide name, institutional affiliation, e-mail address of all protocol authors; provide physical mailing address of corresponding author	YES
Contributions	3b	Describe contributions of protocol authors and identify the guarantor of the review	YES
Amendments	4	If the protocol represents an amendment of a previously completed or published protocol, identify as such and list changes; otherwise, state plan for documenting important protocol amendments	N/A
Support:			
Sources	5a	Indicate sources of financial or other support for the review	YES
Sponsor	5b	Provide name for the review funder and/or sponsor	N/A
Role of sponsor or funder	5c	Describe roles of funder(s), sponsor(s), and/or institution(s), if any, in developing the protocol	YES
**INTRODUCTION**			
Rationale	6	Describe the rationale for the review in the context of what is already known	YES
Objectives	7	Provide an explicit statement of the question(s) the review will address with reference to participants, interventions, comparators, and outcomes (PICO)	YES
**METHODS**			
Eligibility criteria	8	Specify the study characteristics (such as PICO, study design, setting, time frame) and report characteristics (such as years considered, language, publication status) to be used as criteria for eligibility for the review	YES
Information sources	9	Describe all intended information sources (such as electronic databases, contact with study authors, trial registers or other grey literature sources) with planned dates of coverage	YES
Search strategy	10	Present draft of search strategy to be used for at least one electronic database, including planned limits, such that it could be repeated	YES

For peer review only - http://bmjopen.bmj.com/site/about/guidelines.xhtml

Study records:			
Data management	11a	Describe the mechanism(s) that will be used to manage records and data throughout the review	YES
Selection process	11b	State the process that will be used for selecting studies (such as two independent reviewers) through each phase of the review (that is, screening, eligibility and inclusion in meta-analysis)	YES
Data collection process	11c	Describe planned method of extracting data from reports (such as piloting forms, done independently, in duplicate), any processes for obtaining and confirming data from investigators	YES
Data items	12	List and define all variables for which data will be sought (such as PICO items, funding sources), any pre-planned data assumptions and simplifications	YES
Outcomes and prioritization	13	List and define all outcomes for which data will be sought, including prioritization of main and additional outcomes, with rationale	N/A
Risk of bias in individual studies	14	Describe anticipated methods for assessing risk of bias of individual studies, including whether this will be done at the outcome or study level, or both; state how this information will be used in data synthesis	N/A
Data synthesis	15a	Describe criteria under which study data will be quantitatively synthesised	N/A
	15b	If data are appropriate for quantitative synthesis, describe planned summary measures, methods of handling data and methods of combining data from studies, including any planned exploration of consistency (such as I^2, Kendall's τ)	N/A
	15c	Describe any proposed additional analyses (such as sensitivity or subgroup analyses, meta-regression)	N/A
	15d	If quantitative synthesis is not appropriate, describe the type of summary planned	YES
Meta-bias(es)	16	Specify any planned assessment of meta-bias(es) (such as publication bias across studies, selective reporting within studies)	N/A
Confidence in cumulative evidence	17	Describe how the strength of the body of evidence will be assessed (such as GRADE)	N/A

From: Shamseer L, Moher D, Clarke M, Ghersi D, Liberati A, Petticrew M, Shekelle P, Stewart L, PRISMA-P Group. Preferred reporting items for systematic review and meta-analysis protocols (PRISMA-P) 2015: elaboration and explanation. BMJ. 2015 Jan 2;349(jan02 1):g7647.

For peer review only

Supplementary File 2: Search strategy for Pubmed using the E-utilities

The search on Pubmed was done by running the E-utilities software (1) from NCBI in a shell script (see below “retrieve_guidelines_pubmed.sh”) developed specifically for this project. The combination of the search results into excel tables was done with the R software and two excel related R-packages (see below “CVD_guidelines_search.R”) (2,3,4)

The keyword strings used in this search were:

“CVD cardiovascular diseases”
“HF heart failure”
“CAD coronary artery disease”
“CVA cerebrovascular accident stroke”
“PAD peripheral artery disease”
“Aortic disease”
“diabetes”
“dyslipidemia”
“HT hypertension”

Pubmed search term per keyword string:

(((guideline[Title]) OR guideline[Publication Type]) AND (keywordString)) AND (("2012"[Date - Publication] : "2023/01/05"Date - Publication]))

Example: For the keyword string “CAD coronary artery disease “, The search query would be:

(((guideline[Title]) OR (guideline[Publication Type])) AND (CAD coronary artery disease)) AND (("2012"[Date - Publication] : "2023/01/05"[Date - Publication]))

Esearch command for each keyword string:

```
esearch -db pubmed -query "((guideline[Title]) OR guideline[Publication Type]) AND ($KeywordString)" -datetype PDAT -mindate 2012 -maxdate 2023/01/05
```

This is the equivalent of the manual pubmed search automatized in a bash code (mac-zsh). Only pmids are retrieved using efetch and the parameter: “-format uid”.
Pmids are then sorted for uniqueness using bash. The code then loops over all unique pmids to store publication details into tab-delimited text files using efetch and xtract commands.

Find the original codes attached to this document:

1. *“retrieve_guidelines_pubmed.sh”*
2. *“CVD_guidelines_search.R”*

For peer review only

Code 1: retrieve_guidelines_pubmed.sh

```
#!/bin/zsh

#this code works best on macintosh zsh terminal, to try it on another
shell, replace #!/bin/zsh by #!/bin/sh in the shebang line 1.

#$1 is a text file with keywords strings like "HF heart failure"
#$2 is the start year and $3 is the end year for the search formats:
YYYY or YYYY/MM or YYYY/MM/DD

#lauch code command for 2012-2023/01/05 full search:
#./retrieve_on_keyword_opti.sh keywords.txt 2012 2023/01/05

#the code will create and fill up a set of text and csv files for the
search results.

Lines=$(cat $1|wc -l|sed 's/ //g' |cut -f1) #number of keywords
Date1=$(echo $2 | sed 's/\//_/g'|cut -f1) #reformat dates with
underscores
Date2=$(echo $3 | sed 's/\//_/g'|cut -f1)

PMIDsfile=PMIDs_allkeywords_"$Date1"_to_"$Date2".txt
PMIDKEYSfile=pmid_keys_"$Date1"_to_"$Date2".csv
cat pmid_keys_header.csv > $PMIDKEYSfile

for l in {1..$Lines} #for loop over all keyword strings
do
    Keyword=$(cat $1| head -$l | tail -1)

    echo $l: $Keyword
    if [ -f "$Keyword"_"$Date1"_to_"$Date2"_pmids.txt ];
    then
        rm "$Keyword"_"$Date1"_to_"$Date2"_pmids.txt
    fi
    #from the initial esearch, only pmids are stored into a text file
    esearch -db pubmed -query "((guideline[Title]) OR
guideline[Publication Type]) AND ($Keyword)" -datetype PDAT -mindate $2
-maxdate $3| efetch -format uid
>>"$Keyword"_"$Date1"_to_"$Date2"_pmids.txt

    cat "$Keyword"_"$Date1"_to_"$Date2"_pmids.txt >>tmp1.txt

    for k in $(cat "$Keyword"_"$Date1"_to_"$Date2"_pmids.txt)
    do
        echo "$k,$Keyword" >> tmp_pmid_keys.csv
    done
done

#pmids are now sorted for uniqueness
cat tmp1.txt |cut -f1 | sort -dhu | awk 'NR>1'>tmp2.txt
cat tmp_pmid_keys.csv| sort -k1,1 -u | awk 'NR>1'>>$PMIDKEYSfile
cat tmp2.txt | sort -dhu | awk 'NR>1'>$PMIDsfile
rm tmp1.txt
rm tmp2.txt
```

For peer review only

```
rm tmp_pmid_keys.csv

#step2

Detailsfile=Details_Publications_"$Date1"_to_"$Date2".txt
DOIsfile=DOIs_Publications_"$Date1"_to_"$Date2".txt
Abstractsfile=Abstracts_Publications_"$Date1"_to_"$Date2".txt
Typefile=Pub_types_"$Date1"_to_"$Date2".txt

cat header_opti.txt >$Detailsfile
cat DOIheader.txt >$DOIsfile
cat Typeheader.txt >$Typefile
cat abstractsheader.txt >$Abstractsfile
pLines=$(cat $PMIDsfile|wc -l|sed 's/ //g' |cut -f1)

echo "running for $pLines entries"

for p in {1..$pLines} #for loop over all unique pmid to store
publication details into tab delimited text files
do
    PMID=$(cat $PMIDsfile| head -$p | tail -1)

    if [ $(expr $p % 50) -eq 0 ]
    then
        echo "$p over $pLines"
    fi
    #efetch efficiency depends a lot on internet connexion speed and
stability
     efetch -db pubmed -id $PMID -format xml > temp.xml
    cat temp.xml| xtract -pattern PubmedArticle -def "-" -element
MedlineCitation/PMID PubDate/Year PubDate/Month PubDate/Day
PubDate/MedlineDate DateRevised/Month Article/ArticleTitle
Article/VernacularTitle Journal/Title JournalIssue/Volume
JournalIssue/Issue Pagination/MedlinePgn Abstract
MedlineJournalInfo/Country -block Author -tab ", "  -sep " " -element
LastName,Initials >> $Detailsfile

    cat temp.xml | xtract -pattern PubmedArticle -def "-" -element
MedlineCitation/PMID -block Article -tab "; "  -sep "; " -element
ELocationID@EIdType ELocationID >>$DOIsfile

    cat temp.xml | xtract -pattern PubmedArticle -def "-" -element
MedlineCitation/PMID -block Article -tab "; "  -sep "; " -element
PublicationTypeList/PublicationType >>$Typefile

     echo "$(cat temp.xml | xtract -pattern PubmedArticle -def "-" -
element MedlineCitation/PMID -block Article -tab "; "  -sep "; " -def
"-"  -element Abstract/AbstractText | tr -d '\n')\n" >>$Abstractsfile
done

echo "its over";
rm temp.xml
```

For peer review only

Code 2: CVD_guidelines_search.R

```
#You must first run the shell script "retrieve_guidelines_pubmed.sh"

library(xlsx)
library(readxl)

fileversion<-"_v06"
period<-"2012_to_2023/05/01"
filesLocation<-"~/Documents/oneshots/Litterature_search/"

pubmed<-
read.delim(paste0(filesLocation,"Details_Publications_",period,".txt"))
dois<-
read.delim(paste0(filesLocation,"DOIs_Publications_",period,".txt"))
abstracts<-
read.delim(paste0(filesLocation,"Abstracts_Publications_",period,".txt"
))
keys<-read.csv(paste0(filesLocation,"pmid_keys_",period,".csv"))
types<-read.delim(paste0(filesLocation,"Pub_types_",period,".txt"))

getDOI<-function(Elo){
  a<-paste0(strsplit(Elo,split="; ")[[1]],"")

  if (length(a)==2){
    return(paste0(a[2][a[1]=="doi"][1],""))
  }
  if (length(a)>2){
    b<-matrix(a,ncol=2)
    return(paste0(b[,2][b[,1]=="doi"][1],""))
  }
  else{
    return ("")}
}

dois$DOI<-sapply(dois$ELocationIDs, function(x) getDOI(x))
dois$DOI[dois$DOI!=""]<-paste0("http://doi.org/",dois$DOI[dois$DOI!=""
&!is.na(dois$DOI)])

abstracts$depression<-sapply(abstracts$Abstract,function(x)
grepl("depress",x,ignore.case = T))
abstracts$psych<-sapply(abstracts$Abstract,function(x)
grepl("psych",x,ignore.case = T))
abstracts$mental<-sapply(abstracts$Abstract,function(x) grepl(" mental
",x,ignore.case = T)|grepl(" mentally ",x,ignore.case = T))

pubmed$keywords<-
sapply(pubmed$PMID,function(x)paste(keys$Keyword[keys$PMID==x],collapse
=", "))

if(identical(pubmed$PMID,dois$PMID)){
  pubmed$DOI<-dois$DOI
  pubmed$DOI[pubmed$DOI=="http://doi.org/NA"]<-""
}
```

For peer review only

```
if(identical(pubmed$PMID,abstracts$PMID)){
  pubmed$depression<-abstracts$depression
  pubmed$psych<-abstracts$psych
  pubmed$mental<-abstracts$mental
  pubmed$Abstract[abstracts$Abstract!=""]<-"accessible"
}
if(identical(pubmed$PMID,types$PMID)){
  pubmed$Publication_types<-types$Publication_types
}

pubmed[,"Relev/NR = 1/0 ?"]<-character(nrow(pubmed))
pubmed<-pubmed[,c(1:6,ncol(pubmed),7:(ncol(pubmed)-1))]

write.xlsx(pubmed,paste0(filesLocation,"litterature_on_CVD_and_MH_Every
thing_",period,fileversion,".xlsx"),sheetName =
paste0("Pubmed_",period),showNA = F,row.names = F)
write.xlsx(pubmed[pubmed$depression|pubmed$psych|pubmed$mental,],paste0
(filesLocation,"litterature_on_CVD_and_MH_Everything_",period,fileversi
on,".xlsx"),sheetName = paste0("Pubmed_PSY",period),showNA =
F,row.names = F,append = T)

selection<-c("PMID",   "Year",    "Relev/NR = 1/0 ?",    "Title",
      "Journal",  "DOI",      "Publication_types")

write.xlsx(pubmed[,selection][grepl("guideline",pubmed$Publication_type
s, ignore.case = T) & pubmed$VernacularTitle=="-
",],paste0(filesLocation,"litterature_on_CVD_trimed_",period,fileversio
n,".xlsx"),sheetName = paste0("Guidelines_PubType_ENG"),showNA =
F,row.names = F)
write.xlsx(pubmed[,selection][!grepl("guideline",pubmed$Publication_typ
es, ignore.case = T) & pubmed$VernacularTitle=="-
",],paste0(filesLocation,"litterature_on_CVD_trimed_",period,fileversio
n,".xlsx"),sheetName = paste0("Guidelines_Title_ENG"),showNA =
F,row.names = F,append=T)
write.xlsx(pubmed[,selection][pubmed$VernacularTitle!="-
",],paste0(filesLocation,"litterature_on_CVD_trimed_",period,fileversio
n,".xlsx"),sheetName = paste0("non_English_pubs"),showNA = F,row.names =
F,append=T)
```

For peer review only

References

1. Sayers E. The E-utilities In-Depth: Parameters, Syntax and More. 2009 May 29 [Updated 2022 Nov 30]. In: Entrez Programming Utilities Help [Internet]. Bethesda (MD): National Center for Biotechnology Information (US); 2010-. Available from: https://www.ncbi.nlm.nih.gov/books/NBK25499/

2. R Core Team (2022). R: A language and environment for statistical computing. R Foundation for Statistical Computing, Vienna, Austria. URL https://www.R-project.org/

3. Dragulescu A, Arendt C (2020). _xlsx: Read, Write, Format Excel 2007 and Excel 97/2000/XP/2003 Files_. R package version 0.6.5, https://CRAN.R-project.org/package=xlsx

4. Wickham H, Bryan J (2022). _readxl: Read Excel Files_. R package version 1.4.1, https://CRAN.R-project.org/package=readxl

For peer review only
